# Conservation Planning and Reporting Implications of qPCR-based Multi-species eDNA Detection Under EU Environmental Regulations

**DOI:** 10.1007/s00267-026-02503-3

**Published:** 2026-06-06

**Authors:** Simone Giovacchini, Enrico Mirone, Antonia Bruno, Fausto Ramazzotti, Luca Caprotti, Pamela Monaco, Mirko Di Febbraro, Chiara Manfrin, Pushpinder Singh Jamwal, Francesco Belluardo, Andrea Galimberti, Anna Loy

**Affiliations:** 1 Environmetrix Lab, University of Molise, Pesche, Italy; 2 ZooPlantLab, University of Milano-Bicocca, Milan, Italy; 3 National Biodiversity Future Center, Palermo, Italy; 4 Department of Life Sciences, University of Trieste, Trieste, Italy; 5 CNR-IRET, Montelibretti, Italy

## Abstract

European Union (EU) legislation supports the conservation of endangered freshwater species through the Habitats Directive (HD, 92/43/EEC) and the Water Framework Directive (WFD, 2000/60/EC), and the eradication of Invasive Alien Species (IAS) through the Regulation 1143/2014. Periodic monitoring and reporting on the species listed in these regulations are mandatory for EU Member States. Quantitative PCR environmental DNA (qPCR eDNA) based approaches offer a new cost-effective and sensitive tool that could contribute to these monitoring obligations and to spatial conservation planning. We analysed the results from a qPCR eDNA simultaneous survey of 11 endangered species, four IAS, one pathogen fungus and one translocated fish at 53 central-Italian freshwater sites to produce three scores that could be used to prioritize areas of intervention based on the cumulative presence of native and alien species and their prevalence and co-occurrence. For each site, we proposed a Prioritizing Protection Score (PPS), a Prioritizing Eradication Score (PES), and a Benthic Invertebrates and Fish Score (BIFS) in accordance with HD, Regulation 1143/2014, and WFD. PPS prioritized 39 sites eligible for Natura 2000 designation to achieve the EU target of 30% protected land, PES identified 22 sites for IAS eradication and management, whereas BIFS highlighted 33 sites where freshwater evaluations can benefit from eDNA surveys. Results from the qPCR eDNA survey also revealed 53 new grid cells of occurrence of IAS and species listed in Annexes II, IV and V of HD that will contribute to the next reporting for both the HD and the IAS Regulation.

## Introduction

Freshwater ecosystems are among the most threatened globally, impacted by multiple anthropogenic stressors (Sanders et al., 2024). Habitat loss, artificial barriers, water drainage, overexploitation, and pollution have led to a significant decline in numerous freshwater species (Costa et al., 2021; Jaureguiberry et al., 2024). To curb biodiversity loss, in 1992, the International Convention on Biological Diversity (CBD) (https://www.cbd.int/) committed signatory countries to develop national strategies, plans, or programs for the conservation and sustainable use of biological diversity.

Accordingly, in 2000, the European Union (EU) adopted Directive 92/43/EEC, commonly known as the Habitats Directive (HD). This Directive requires all Member States to commit to preserving natural habitats as well as wild fauna and flora of Community interest in a favorable conservation status through the implementation of conservation actions or restoration activities. Up to 91 freshwater species have been recognized as species of Community interest and listed in HD Annexes II, IV, and V, entailing the designation of Special Areas of Conservation (SACs) (Annex II), the strict protection (Annex IV), and the regulation of a sustainable exploitation of the species (Annex V). To monitor the effectiveness of these conservation measures, each Member State is required to report on the distribution, conservation status, and population trend of these species every 6 years. Consequently, surveillance monitoring programs are essential for achieving the objectives of the HD (Zettler and Ellwanger et al., 2018; Jueg 2007).

Moreover, for species listed in Annex II and Habitats in Annex I, the Directive mandates the establishment of a network of protected areas composed of SACs, which, together with the Special Protection Areas designated under the Birds Directive (2009/147/EC), collectively form the EU Natura 2000 Network (N2KN hereafter). The N2KN has been recognized as a significant contributor to the achievement of the EU Biodiversity Strategy to 2030 (https://environment.ec.europa.eu/strategy/biodiversity-strategy-2030_en), as part of the Kunming-Montreal Global Biodiversity Framework adopted in 2022 during the 15th COP of the CBD (https://www.cbd.int/gbf). Moreover, in line with these goals, in 2024 the European Commission adopted a Nature Restoration Law, which mandates the Member States to expand protected area coverage by at least 30% of the European Union territory. Protected areas include the N2KN, currently covering 18.6% of the EU’s land area and 9% of its marine territory, encompassing more than 27,000 SACs (EEA 2022).

The conservation of freshwater species that are endangered (i.e. broadly recognised as in danger of extinction, although not classified under the specific IUCN category of ‘Endangered’) listed under the Habitats Directive often depends on the quality of both aquatic ecosystems and terrestrial ecosystems directly depending on them. In this context, the European Water Framework Directive 2000/60/EC (hereafter WFD) made a crucial contribution to the conservation of freshwater habitats and species (Weigelhofer et al. 2020), as its primary objective is to achieve good chemical and ecological status for all rivers, lakes, and transitional and coastal waters in the EU. Specifically, after a long process of intercalibration among all Member States and the EU Ecological Status (ECOSTAT) Working Group to harmonize national methods to classify waters, the ecological status of water bodies is assessed using indices primarily derived from biological surveys of aquatic flora, benthic invertebrates, and fish communities (Buffagni and Erba 2007; Macchio et al. 2017; Hering et al. 2018). These indices contribute to the implementation of river basin management plans (Birk et al. 2012) through which the WFD is directly linked to the HD, as their objectives overlap. Each river basin district must identify areas requiring special protection under specific Community legislation for the conservation of habitats and species directly depending on water (Art. 4, Art. 6, and Annex VII), highlighting the synergies of the WFD and HD, which are often overlooked (Bolpagni et al. 2017).

Thus, as conservation measures identified under HD are transferred toward the river management plans under the WFD, the latter are developed in part also in response to specific threats affecting endangered habitats and species. Among the most significant threats to biodiversity are invasive alien species (IAS) (Dextrase and Mandrak, 2006; Francis, 2012). Indeed, IAS are considered one of the primary drivers of biodiversity loss worldwide (Dueñas et al., 2021; IPBES, 2023), particularly in freshwater ecosystems (Meira et al., 2019). Biological invasions alter ecosystem composition and structure, reducing the diversity of native communities and consequently diminishing ecosystem functions, resilience, and resistance (IPBES 2023).

Compared with other continents, Europe has experienced the highest number of invasions by freshwater alien species—such as fish, crustaceans, and molluscs (IPBES, 2023), with Italy as one of the most affected countries (Gherardi et al., 2008; Magliozzi et al., 2020), where the number of introduced species recently exceeded that of native species (Lorenzoni et al., 2019).

To prevent new introductions and manage the spread of IAS, the EU adopted Regulation 1143/2014 (Genovesi et al., 2015). A key component of this regulation is the establishment of a surveillance system (Pagad et al., 2015). Under this framework, Member States are required to implement a monitoring system for the early detection of 88 IAS of Union Concern (as amended by Regulation 2022/1203). Additionally, every 6 years, Member States must update the distribution data of these IAS and evaluate the effectiveness of rapid eradication measures (Article 24 of Regulation 1143/2014). Concurrently, management measures should be applied to the IAS that have become widespread. Accordingly, spatial prioritization is strongly recommended—focusing on eradicating IAS in areas where they have recently appeared and mitigating their impact in regions that are heavily colonized. However, monitoring multiple species across various taxa is time-consuming and requires substantial expertise and financial resources, which can impede the implementation of effective monitoring programs.

In this complex European system of monitoring obligations, environmental DNA (eDNA) represents a promising and cost-effective tool for the simultaneous detection of multiple species across diverse taxa (Taberlet et al., 2018). Specifically, eDNA-based approaches have proven particularly effective in monitoring freshwater communities and detecting rare and elusive species that often remain undetected using traditional field surveys (Huang et al., 2022). Accordingly, eDNA could serve as an effective complementary tool to fulfill the monitoring obligations under the HD, facilitate the early detection of invasive alien species of Union Concern under Regulation 1143/2014, and support ecological status assessment under the WFD (Giovacchini et al., unpublished; Thomsen et al., 2012; Leese et al., 2018).

Detection of multiple species through eDNA typically employs a metabarcoding approach. This method involves sequencing standardized genomic regions common to many metazoans to identify the composition of a biotic community at broad taxonomic scales (Taberlet et al., 2018). Although advances in eDNA metabarcoding over the past decade have improved taxonomic resolution to the species level, the single-species approach based on quantitative PCR (qPCR) remains a more sensitive alternative (Harper et al., 2018). This increased sensitivity is particularly important for detecting rare, endangered species and for identifying invasive species at the early stages of colonization (Hernandez et al., 2020; Xia et al., 2021).

However, qPCR-based eDNA approaches are rarely used to simultaneously detect multiple species (Wozney and king et al., 2022; Minegishi et al., 2023; Tsuji et al., 2018; Wilson 2017), and are even less commonly applied to broad taxonomic inventories (Leblanc et al., 2020; Seymour et al., 2018; Thomsen et al., 2012).

With this study, we examine the conservation and management implications of an eDNA-based simultaneous detection of both endangered and alien taxa occurring at each site in freshwater bodies of Central Italy (Giovacchini et al., 2026a; Loy et al., 2023). We explored the opportunities of eDNA-based techniques to detect species of conservation concern, contribute to EU reporting obligations following the HD and IAS regulation, and eventually evaluate the ecological status of sampling sites following the WFD classification. Specifically, our objectives were to: (i) develop three indices to classify the sampling sites based on eDNA occurrence and co-occurrence of native endangered and alien species; (ii) provide a scoring system to identify priority conservation areas in accordance with the two European regulations (HD, IAS Regulation); (iii) contribute to the upcoming reporting cycle of the Habitats Directive by updating the distribution of species listed in Annexes II, IV, and V; (iv) to provide early warning of new IAS of Union Concern and update their distribution in line with Regulation 1143/2014; and (v) to integrate eDNA into routine monitoring under the WFD.

## Methods

### Species Selection

The species were selected based on their legal status and monitoring obligations under the European Union regulations (Table 1). Specifically, we considered species listed in Annex II, IV, and V of the HD 92/43/EEC, IAS of Union Concern under regulation 1143/2014 (Flitcroft et al., 2025), and IAS that may pose a severe threat to the endangered ones. In this context, eDNA detections could support reporting obligations under both regulatory frameworks and, for species listed in Annex II of the HD, help identify potential new Natura 2000 sites, thereby contributing to the targets of the Nature Restoration Regulation (1991 /2024/EC), which aims to protect 30% EU territory by 2030.

**Table 1:** List of species used in the study and the respective legislation to which they refer

Clade	Species	Synonym	Species included in	Lentic /Lotic sampling site
Bivalves	*Unio mancus*	*U. elongatulus*	HD Annex V IMG for WFD (benthos)	both
Gastropods	*Vertigo spp*.	*V. angustior* *V. moulinsiana*	HD Annex II	both
Crustaceans	*Austropotamobius italicus* ^*#*^	*A. pallipes*	HD Annex II, IV IMG for WFD (benthos)	lotic
Osteichthyes	*Alosa fallax*	–	HD Annex II, IV IMG for WFD (fish)	lotic
Osteichthyes	*Squalius lucumonis* ^*#*^	–	HD Annex II IMG for WFD (fish)	lotic
Amphibians	*Bombina pachypus* ^*#*^	–	HD Annex II, IV	both
Amphibians	*Triturus carnifex* ^*#*^	–	HD Annex II, IV	lentic
Amphibians	*Lissotriton italicus* ^*#*^	–	HD Annex IV	lentic
Mammals	*Mustela putorius*	–	HD Annex V	both
Mammals	*Lutra lutra*	–	HD Annex II, IV	lotic
Bivalves	Dreissena polymorpha *	–	IUCN 100 of the World’s Worst IAS	both
Crustaceans	Faxonius limosus *	*Orconectes limosus*	IAS of the Union concern	both
Crustaceans	Procambarus clarkii *	–	IAS of the Union concern	both
Osteichthyes	Pseudorasbora parva *	–	IAS of the Union concern IMG for WFD (fish)	both
Osteichthyes	Padogobius bonelli *	*P. martensii*	introduced outside its native range IMG for WFD (fish)	lotic
Amphibians	Lithobates catesbeianus *	–	IAS of the Union concern IUCN 100 of the World’s Worst IAS	lotic
Fungi	Batrachochytrium dendrobatidis *	–	IUCN 100 of the World’s Worst IAS	both

Alien invasive species are marked with an asterisk (*). Endemic species are marked with a hash symbol (#)

*HD* Habitats Directive. *IAS* Invasive Alien Species. *IMG for WFD* Italian Monitoring Guidelines to assess the ecological status of water bodies for the Water Framework Directive

Species selected include ten species listed in Annexes II, IV, and V of the HD, five IAS of Union concern, the translocated fish *Padogobius bonelli*, and the chytrid pathogen *Batrachochytrium dendrobatidis*, which threaten native amphibians worldwide (Beranek et al., 2021) and are included in the IUCN 100 worst invasive species impacting biodiversity at a global scale (Lowe et al., 2000).

Six species are also included in the Italian monitoring guidelines to assess the ecological status of national freshwater bodies according to the Water Framework Directive (2000/60/EC) as species sensitive to water quality or contributing to affect the composition of native fish communities. Specifically, *Unio mancus* and *Austrapotamobius italicus* contribute to the STAR_ICMi index implemented for benthic invertebrate communities (Armitage et al., 1983; Buffagni and Erba, 2007), whereas two native fish (*Alosa fallax* and *Squalius lucumonis*) and two IAS fish (*Padogobius bonelli*, *Pseudorasbora parva*) are included in the NISECI index implemented for fish communities (Macchio et al., 2017).

### Study Area and Survey Design

**Fig. 1:**
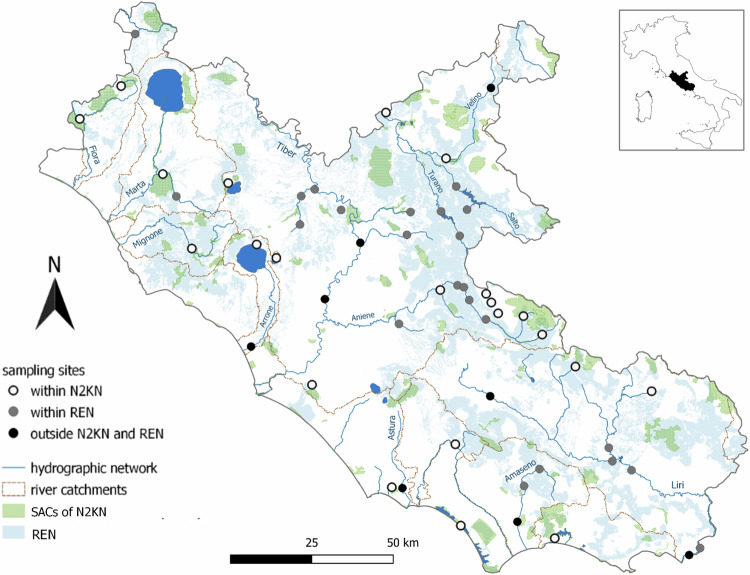
The study area (Latium region) showing sampling sites, river catchments, Special Areas of Conservation included in Natura 2000 Network (SACs of N2KN) and the Regional Ecological Network (REN) of Latium Region

The study area corresponds to the Latium administrative region, located in Central Italy along the Tyrrhenian side (Fig. 1). The administrative regions are, together with national parks and autonomous provinces, the institutional entities with responsibility for regulation, monitoring and EU reporting. Latium is characterized by highly heterogeneous landscapes, from hills to volcanic plateaus, from inland mountains to piedmont areas, lowlands and seacoast. The region is intersected by the Tiber, the main river basin of Central Italy (405 km in length). The remaining river catchments are smaller and mainly located near the coast. Natural lakes are set in the northern part of the region and along the southern coasts, whereas two artificial reservoirs are found in the inland mountain ranges. This high heterogeneity of landscapes hosts a high diversity of animal and plant communities (Balletto et al., 2010). The region also represents a biogeographic parapatric contact zone between species endemic to south-central Italy and vicariant species more widely distributed in Europe (Iannella et al., 2017). Species endemic to the Apennines include the Italian crayfish *Austropotamobius italicus*, the Apennine yellow-bellied toad *Bombina variegata pachypus*, and the Tuscany stream chub *Squalius lucumonis* (Brusconi et al., 2008; Canestrelli et al., 2013; Tancioni et al., 2013). At the center of the study area is the large metropolitan city of Rome, with a human population of more than 4 million, which has contributed to the spread of several IAS (Monaco, 2014; li et al., 2024).

According to our aims, the sampling design was mainly addressed to Nature 2000 sites and WFD sampling sites. We selected 53 sampling sites located in 14 lentic and 39 lotic water bodies that were part of the routine regional monitoring network for the WFD, covering a wide range of freshwater habitats, from rivers, creeks, channels, to lakes, reservoirs, and ponds (Giovacchini et al., 2026a; Mirone et al., 2024). They were located both within (*n* = 23) and outside (*n* = 30) the N2KN of Special Areas of Conservation, and the Regional Ecological Network established by the regional administration of Latium to connect protected areas and guarantee wildlife movements among them (Scalisi et al., 2011).

Each site was sampled twice, with one replicate performed in summer 2021 and one in spring 2022. Environmental samples consisted of 3 L of water collected through sterile bottles and filtered within 24 h through 8 µm MCE prefilters and 0.22 µm MCE filters (Merck-Millipore, Burlington, USA) using an electric vacuum pump (KNF Laboport, Neuberger GmbH, Germany). All filters were immediately stored at –20 °C until DNA extraction (Mirone et al., 2024).

### Laboratory Analyses

For seven species out of 17, we used published primers and probes, including *Batracochytrium dendrobatidis* (Canessa et al., 2017), *Dreissena polymorpha* (Amberg et al., 2019), *Austropotamobius italicus* (Manfrin et al., 2022a), *Alosa fallax* (Antognazza et al., 2019) *Pseudorasbora parva* (Manfrin et al., 2022b), *Lithobates catesbeianus* (Sanz et al., 2023), and *Lutra lutra* (Park et al., 2011). For the remaining 11 species, new species-specific primers and probes were designed (Loy et al., 2023). Details on assays design and validation are reported in Mirone et al. (2024), and are summarized here. International molecular databases (BOLD Systems, GenBank, NCBI) were explored to identify the most suitable molecular markers accounting for both intra- and interspecific genetic variability, phylogenetic proximity, and potentially syntopic taxa (Klymus et al., 2020).

Reference DNA sequences for each candidate marker were aligned using the MAFFT online tool (Katoh et al., 2009). Sequence evaluation included assessment of missing data, polymorphic sites, mismatches between taxonomic classification and molecular assignment, and sequence length (Wilcox et al., 2013). Candidate primers and probes were identified within suitable nucleotide regions using BioEdit (Hall 1999).

The oligonucleotides were then subjected to three validation steps (in silico, in vitro, and in situ) following Thalinger et al. (2021). Potential co-amplifications were minimized by selecting primers and probes with annealing temperatures between 55 and 62 °C, probe melting temperature approximately 4 °C higher than that of primers, GC content around 50% within binding regions, primers of approximately 18–22 bp, probe length of about 25 bp, and amplicon length of approximately 150 bp. Primer pairs meeting these physico-chemical criteria were further tested using Primer-BLAST.

Primers and probes passing the in silico validation step were subsequently tested in vitro on tissue samples of the target species. PCR reactions were performed using genomic DNA extracts as templates and primers only. Details on optimal annealing temperatures are provided in Mirone et al. (2024). Agarose gel electrophoresis (2%) was used to verify the presence of an amplicon corresponding to the expected target fragment size (Wilcox et al., 2015). Amplicons were then subjected to Sanger sequencing to confirm the correct assignment of the reference sample to the target genetic region and species of interest using NCBI’s BLAST tool (Altschul, 1997; Ye et al., 2012).

In the final in situ validation step, primers and probe combinations were used to set up qPCR assays with TaqMan technology (Bylemans et al., 2016). Each run included (i) a PCR positive control consisting of genomic DNA extracts from the target species; (ii) eDNA extracts derived from water samples collected in two sites of ascertained presence of the species (iii) eDNA extracts from water samples from two sites of ascertained absence of the species. To verify the absence of contamination by targeted or untargeted DNA, PCR negative controls were included in triplicate during reaction set-up steps (Klymus et al., 2020; Karlsson et al., 2022; Thalinger et al., 2021). Amplification efficiency (E) and limit of detection (LOD) were calculated, and standard curves were generated using tenfold serial dilutions of quantified positive tissue controls (Giovacchini et al., 2026a; Loy et al., 2023; Mirone et al., 2024). E ranged from 82.2% to 191.9%, and LOD values were between 4,00 × 10^–7^ and 2,90 × 10^–3 ^ng. These parameters were used to evaluate assay performance during in vitro validations and indicated high sensitivity of the primers and probes. All assays showed no amplification in the field negative controls (i.e., environmental samples of confirmed absence of the target species) and successfully amplified all the environmental samples collected at sites with confirmed species presence.

DNA was extracted from filters using the DNeasy PowerSoil Pro kit (Qiagen, Venlo, NL), diluted in 75 μl bidistilled Miili-Q sterile water and preserved at –21 °C. Each sampling site was tested with just a subset of the 17 species-specific assays, ranging from 5 to 12 species, based on the number of native and alien species potentially occurring at the site according to the criteria of Giovacchini et al. (2026a). Published and newly developed assays of target species were provided by Eurofins Genomics srl (Hamburg, DE) and tested using real-time qPCR with TaqMan technology (StepOnePlus, Applied Biosystems, Waltham, USA). Amplification details can be found in Mirone et al. (2024). The reaction mix comprised 2.35 µl of Milli-Q water, 5 µl of buffer (Eurofins Genomics srl, Hamburg, DE), 0.25 µl of each primer (10 µM), 0.15 µl of dual-labeled Taqman probe (10 2 µM; Eurofins Genomics srl, Hamburg, DE), and 2 µl of extracted DNA. Samples were run in two dilutions (1:1, and 1:10) in three technical replicates, together with a PCR positive control with genomic DNA of target species, a PCR negative technical control to verify the absence of contamination using DNA of untargeted species, and an environmental positive and negative control (water samples collected in sites of ascertained presence and absence of target species) (Klymus et al., 2020; Karlsson et al., 2022; Thalinger et al., 2021). To verify the correct extraction of eDNA, samples were also tested with a MiFish set of universal 12S rRNA oligonucleotides for fish DNA metabarcoding characterization (Miya et al., 2015). To avoid contamination, we carried out pre and post-amplification phases in separate rooms in a laminar flow cabinet. We considered positive samples when observed in at least two technical replicates, and above the limit of detection, or under it when the number of DNA copies was equal or more than five per reaction (Bruno et al., 2017; Bustin et al., 2009; Ficetola et al., 2015).

### Site Scores

Species eDNA occurrences were overlapped to their known distribution (10 × 10 km cell grids in ETRS LAEA 52 10 projection) provided by the last fourth HD Reporting (Ercole et al., 2021) or by the last 2015–2018 IAS reporting under EU Regulation 1143/2014 (http://www.eea.europe.eu/en/datahub/), or derived from published surveys on IAS (Sarrocco et al., 2012; Canestrelli et al., 2013; Carosi et al., 2021; Grano et al., 2020; Grano, 2022; Zampiglia et al., 2013). Each species was only tested on potential sites of occurrence to optimize the costs of qPCR, thus resulting in different sampling efforts. To account for unbalanced sampling of alien *vs* native sample sizes, we first produced a Native-Alien Prevalence index (NAP) species, as follows:

$${\mathrm{NAP}}\,=\,{{\rm{N}}}_{\rm{r}}\,-\,{{\rm{A}}}_{{\rm{r}}}$$

where

$${{\rm{N}}}_{{\rm{r}}}\,=\,{\rm{n}}.\,\mathrm{native}\,\mathrm{species}\,\mathrm{detected}/{\rm{n}}.\,\mathrm{native}\,\mathrm{tested}$$

$${{\rm{A}}}_{{\rm{r}}}\,=\,{\rm{n}}.\,\mathrm{IAS}\,\mathrm{detected}/{\rm{n}}.\,\mathrm{IAS}\,\mathrm{tested}$$

NAP varies from –1 to 1, where NAP > 0 identifies near-natural areas with prevailing native species, NAP = 0 identifies vulnerable areas where native endangered species (i.e., listed in Annex II, IV, and V of the Habitats Directive) species and IAS co-occur in similar proportions, and NAP < 0 highlights areas heavily colonized by IAS, because their number exceeds that of the native ones. The index also accounts for testing only one category of target species, i.e., only native endangered species or only IAS. So, as an example, if we found three native species where we were looking for eight of them, and one IAS where we tested for just two IAS, the NAP index resulted in equal to –0.125, as a consequence of 3/8-1/2.

Occurrences of species listed in Annex II of the Habitats Directive were then overlapped with SACs in the study area, and the corresponding Standard Data Forms were examined to verify whether the species had already been recorded at the site, i.e., whether the site had also been designated for that species as a primary feature.

**Fig. 2:**
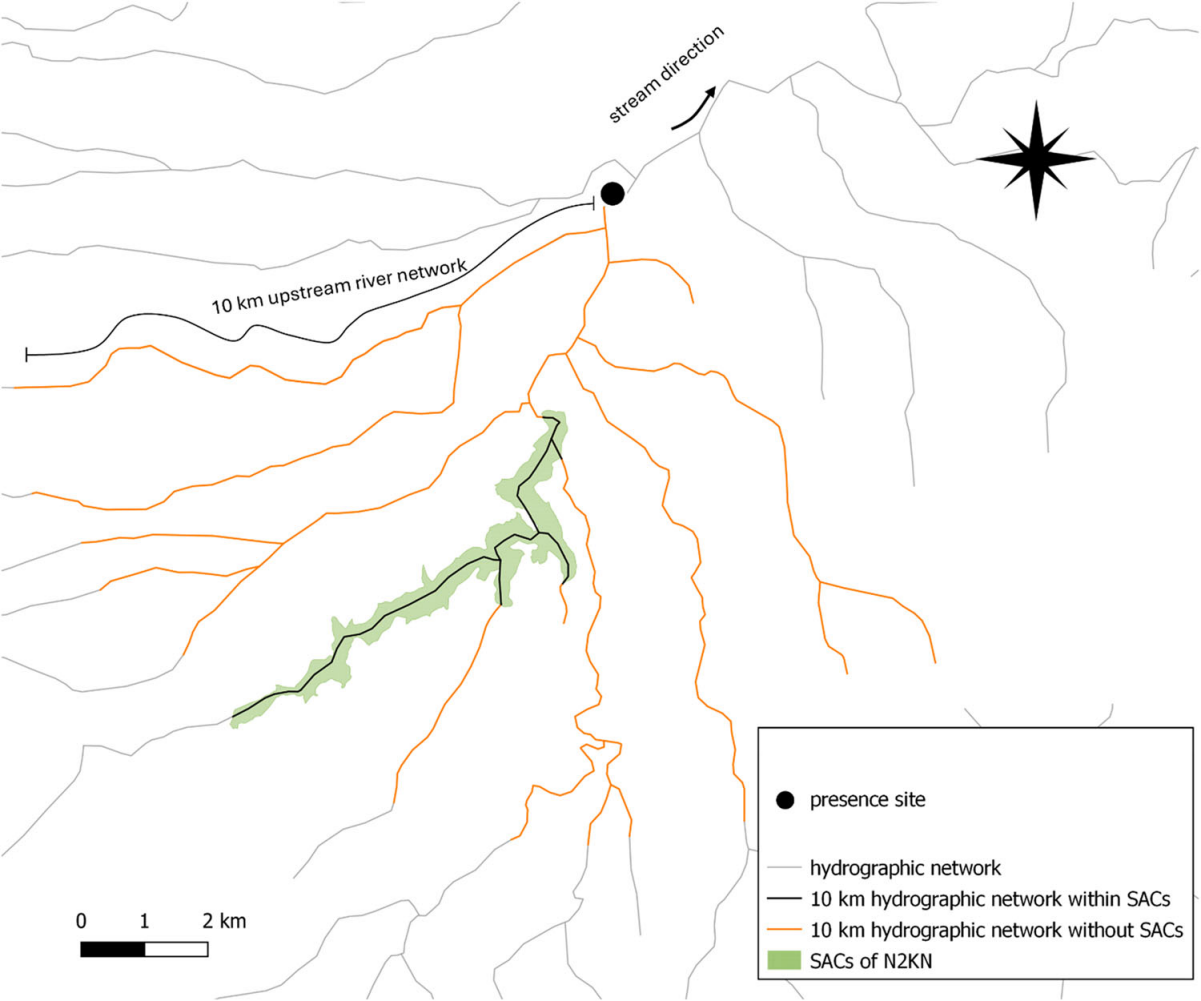
Accounting for eDNA transported from upstream to the sampling sites. Starting from a new occurrence site (green circle) of a species listed in Annex II of the Habitats Directive, we selected 10 km of the upstream river network (hydrographic network highlighted with black and orange colours), and overlapped the Special Areas of Conservation of the Natura 2000 Network (N2KN) along the resulting stream network to reveal if it is covered by N2KN (black) or not (orange)

To account for potential false-positive detections at lotic sites resulting from downstream transport of eDNA (Burian et al., 2021; Deiner et al., 2016; Sepulveda et al., 2020), we introduced a spatial uncertainty factor. Positive detections were extended upstream according to the best available evidence on eDNA persistence and transport distance in running waters (Deiner and Altermatt, 2014; Civade et al., 2016; Deiner et al., 2016; Jane et al., 2015; Padgett-Stewart et al., 2016; Pont et al., 2018; Song et al., 2017; Villacorta-Rath et al., 2021; Wilcox et al., 2016). Since studies based on the collection of a few liters of water reported eDNA transport from 3 to 20 km (Deiner and Altermatt 2014; Civade et al., 2016; Villacorta-Rath et al., 2021), species occurrence was extended upstream by an average distance of 10 km from the sampling site, including tributaries (Fig. 2). The resulting potential area of occurrence was overlapped with the SACs of the N2KN to identify gaps and priority areas for N2KN extension. Considering the unprotected stretches of the river as patches, we assessed the spatial pattern of patches through the measure of three landscape metrics to assess how the potential area of occurrence was fragmented in its current protection status. We assessed: (i) the number of patches (hereafter, patch number); (ii) the length of patches over the total length of the river network occurring in the potential area of occurrence (hereafter, patch density); (iii) the average Nearest Neighbor Distance (NND) of the nearest patch along the river network of the area of potential occurrence (Rutledge, 2003). A higher patch number means that the river network within the potential area of occurrence is fragmented in its protection status with a higher number of patches. Similarly, a high patch density highlights how much the river is unprotected overall, despite its fragmentation in protection status. A higher NND shows that patches are reciprocally positioned far instead of being close. All analyses were performed using QGIS 3.28 (http://qgis.org).

To prioritize conservation areas according to the Habitats Directive, for each site we assigned a priori values based on the presence of species of Community interest within or outside SACs of N2KN and the degree of the fragmentation of the protection status of the area, and we computed a Prioritizing Protection Score (PPS), as follows:

$${\mathrm{PPS}}={\mathrm{NAP}}+({\rm{N}}2{\mathrm{KN}}_{\mathrm{out}}+{\rm{N}}2{\mathrm{KN}}_{\mathrm{in}}+\mathrm{REN})* {\mathrm{patch}}\; {\rm{density}}$$

where

N2KN_out_ = 2, if the site falls outside N2KN,

N2KN_in_ = 1, if the site falls inside N2KN,

REN = 1, if the site falls inside a Regional Ecological Network.

$$\mathrm{PPS}\,\mathrm{is}\,\mathrm{finally}\,\mathrm{normalized}\,\mathrm{from}\,0\,\mathrm{to}\,1.$$

To prioritize areas for IAS eradication, for each site we assigned a priori values, and we computed a Prioritizing Eradication Score (PES), as follows

$${\mathrm{PES}}=|\,{\mathrm{NAP}}-\,({{\rm{N}}2\mathrm{KN}}_{\mathrm{in}}-{\mathrm{co}}\mbox{-}{\mathrm{oc}}_{\mathrm{site}}-{\mathrm{co}}\mbox{-}{\mathrm{oc}}_{\mathrm{river}\,{\mathrm{basin}}})|,$$

where

$${\rm{N}}2{\mathrm{KN}}_{\mathrm{in}}\,=\,1,\,\mathrm{when}\,\mathrm{the}\,\mathrm{site}\,\mathrm{falls}\,\mathrm{within}\,{\rm{N}}2\mathrm{KN},$$

co-oc _site_ = 2, if IAS co-occurred at the same site with the native species they directly threaten (e.g., *B. dendrobatidis* vs *B. pachypus*: Porco et al., 2024),

co-oc _river basin_ = number of native species detected in the river catchment directly threatened by the IAS detected at the sampling site (e.g., 2, if *B. dendrobatidis* is revealed in the same river catchment where *T. carnifex* and *L. italicus were detected at other sites*).

### PES is Finally Normalized from 0 to 1

Finally, to align with the WFD national guidelines, we developed a Benthic Invertebrates and Fish Score (BIFS) to identify areas where the eDNA can assist the WFD as a complementary tool and better inform the assessments on the ecological status of European water bodies. In this case, we only considered benthic invertebrates and fish species contributing to WFD evaluations according to the Italian monitoring guidelines (Armitage et al., 1983; Buffagni and Erba, 2007; Macchio et al., 2017). We overlaid the STAR_ICMi (available for the study area for the years 2021 and 2023, see Fig. 6) and NISECI (available for 2018-2020) indices accessible at https://sira.arpalazio.it/web/guest/cartografia. Following the same classification scores of the WFD, we scored all rivers not reaching a Good ecological status from 3 (Moderate) to 1 (Bad), separately for both STAR_ICMi (WFD_B_) and NISECI evaluations (WFD_F_). Although the site is classified as ‘Not Evaluated’, we consider the presence of endangered species as an indication of an ecological status different from bad. Accordingly, we assigned an intermediate value between 1 and 3. For each sampling site, we computed a *benthic score* as follows:

$${Benthic}\,{score}\,=\,{\mathrm{WFD}}_{{\rm{B}}}\,* \,{\mathrm{number}}\,\mathrm{of}\,{\mathrm{benthic}}\,{\mathrm{invertebrate}}\,{\mathrm{species}}\,{\mathrm{detected}}\,{\mathrm{at}}\,{\mathrm{site}}.$$

A Native-Alien Prevalence index was also calculated on fish (NAP_F_). We obtained a *fish score* according to the following formula:

$${Fish}\,{score}={{\rm{NAP}}}_{{\rm{F}}}{\rm{x}}({{\rm{WFD}}}_{{\rm{F}}}+0.1).$$

We artificially converted all values < 0 of the *fish score* to be the symmetrical values obtained > 0 in order to have a proportionate and linear scale between positive and negative values for all sampling sites matching WFD_F_ and NAP_F_ (i.e., 3_[NAPf]_ x (-1_[WFD]_ + 0.1) = -3.3 is converted to -1.1) (Table 2).

**Table 2:**
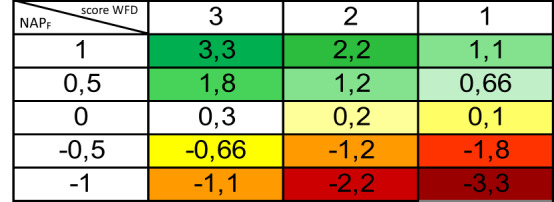
Scoring procedure for sampling sites in which we detected fish species contributing to WFD evaluations according to the Italian monitoring guidelines (see the text for details)

The final BIFS was obtained by summing the *benthic score* and the *fish score*, as follows:

$${\rm{BIFS}}={benthic}\,{score}+{fish}\,{score}$$

and finally normalized from 0 to 1.

## Results

### New Species Occurrences

We obtained 101 species occurrences across 45 sites, including 58 new presence sites for 15 of the 17 target species. New presence data for species included in Annex II, IV and V corresponded to 36 new 10 × 10 km grid cells to be notified in the next (fifth) HD reporting cycle (Online Resource 1-7). We also detected 17 new cells hosting IAS that will contribute to the next 2019–2024 reporting under the EU IAS Regulation (Online Resource 1-7). Moreover, we found 1 to 3 (1.78 ± 0.67 per site) species listed in HD Annex II at 23 sites located outside N2KN (Online Resource 8). Also, four species included in Annex II HD were not reported in the standard forms of the nine Special Areas of Conservation of N2KN (Online Resource 9).

### Species Richness and Co-occurrence

**Fig. 3:**
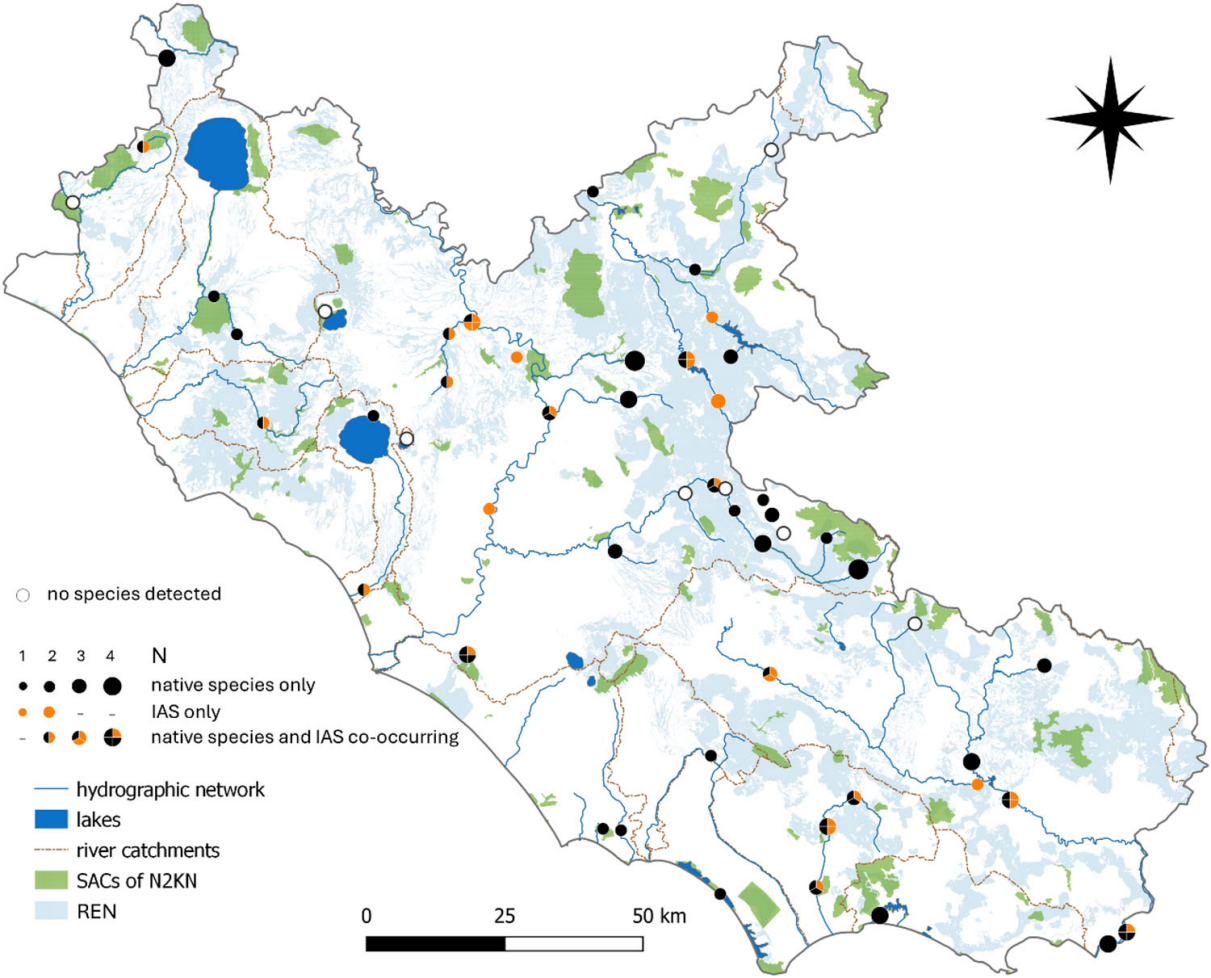
Native protected and alien species richness found within and outside the SACs of the Natura 2000 network. The size of the symbols is proportionate to the total number of species detected at the site (1 to 4)

**Fig. 4:**
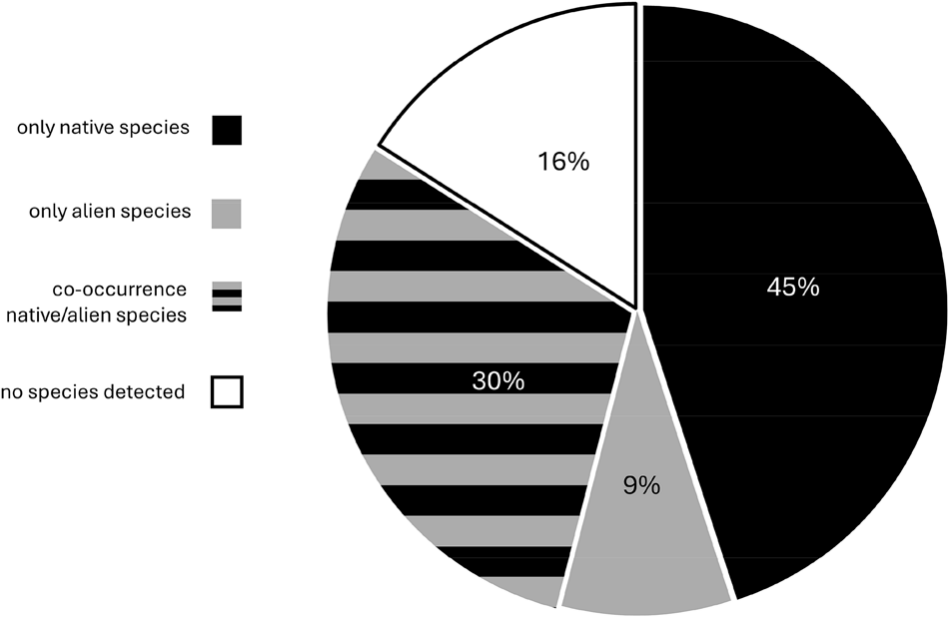
Pie chart showing percentages of sampling sites in which neither native nor alien species were detected, were detected alone, or were detected together in co-occurrence

Only native species were found at 24 sampling sites (45%), ranging from 1 to 4 species per site (Fig. 3). These sites were mainly distributed in mountains and headwater areas. Conversely, only IAS (1 or 2 species) were found at five sites (9%) located along lowland main rivers, channels or artificial reservoirs. Both native species and IAS were found at 16 sites (30%), the former slightly more frequent than the latter (i.e., 27 vs 22) (Fig. 4).

At site scale, at three sites we found the co-occurrence of IAS that directly threaten native endangered species (*B. dendrobatidis* vs *B. pachypus* along River Aniene, *F. limosus* vs *A. italicus* along River Turano, *P. clarkii* vs *Vertigo* sp. at the head of River Fiora in Fig. 3). Moving to the river catchment scale, we identified a total of six IAS directly threatening six native species occurring in the lowland section of the Tiber river, suggesting that additional negative interactions may emerge in the future (*D. polymorpha* vs *U. elongatulus*, *P. clarkii* vs *A. italicus, F. limosus* vs *Vertigo* sp., *L. catesbeianus* vs *B. pachypus, B. dendrobatidis* vs *L. italicus, P. parva* vs *T. carnifex*). Another potential interaction (*B. dendrobatidis* vs *T. carnifex*) was revealed in the Tiber river basin along the main tributary Aniene. A last interaction was assessed in the Garigliano river basin (*P. parva* vs *L. italicus*).

### eDNA Interpolation for Running Water Sites

**Fig. 5:**
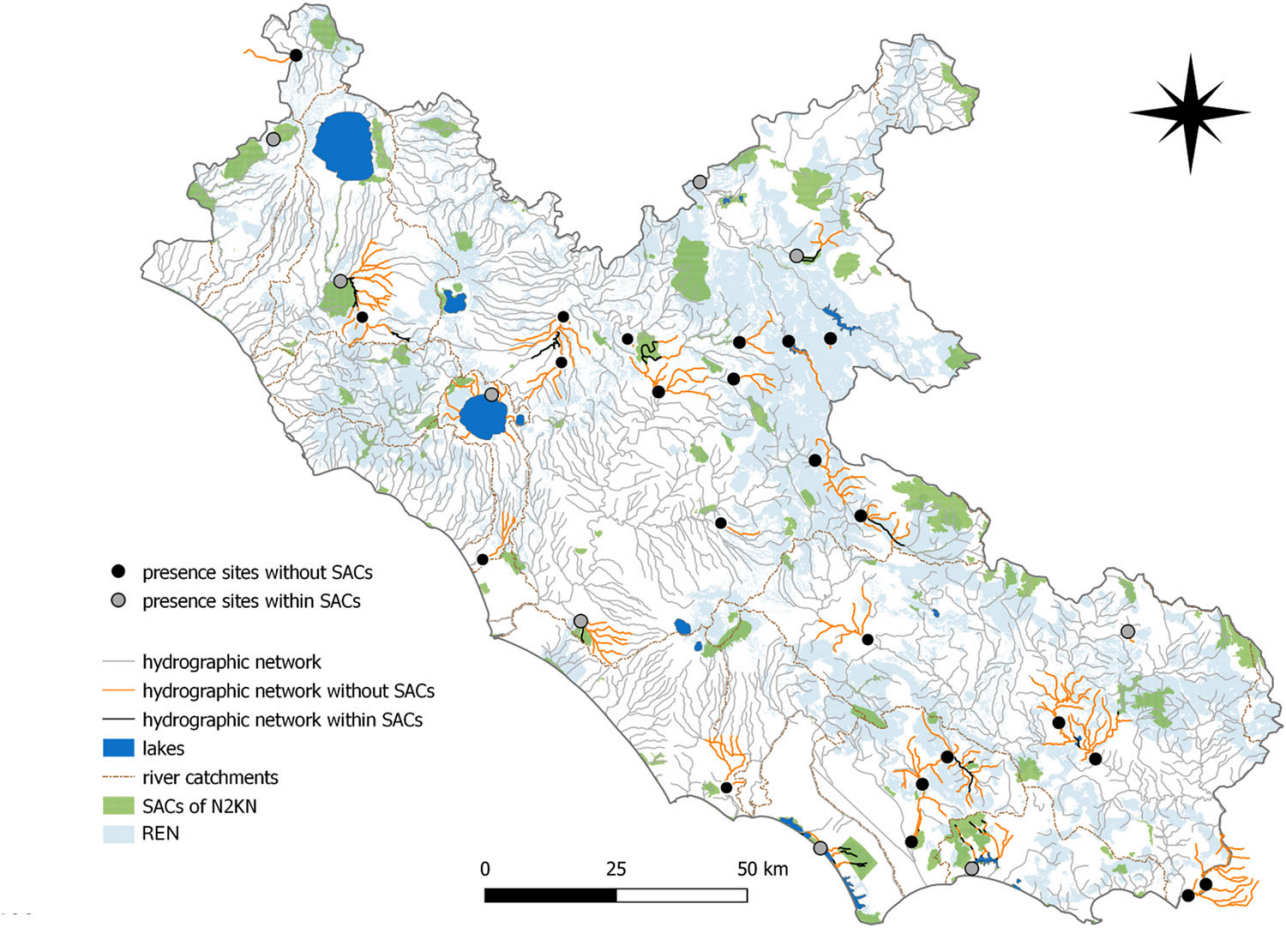
New presence sites of target species listed in Annex II of the Habitats Directive for which the designation of a Special Area of Conservation belonging to the Natura 2000 Network might be required. New presence sites found outside SACs of the Natura 2000 network are shown as black dots, and new presence sites found within SACs of the Natura 2000 network but not reported in the standard forms are shown as grey dots)

New presence sites of target species listed in Annex II of the HD found outside the N2KN generated a cumulative upstream river network (defined as Annex II potential areas) of 808 km, most of which fall in areas without any form of protection (86.1%). Specifically, we found 42 patches of unprotected river stretches dispersed across 23 areas of occurrence of species in HD Annex II (Fig. 5). For each species in HD Annex II, the mean cumulative length of unprotected patches amounted to 30.3 ± 22.4 km (median = 21 km) potential area, with a mean patch density of 0.89 ± 0.2. Single patches had an average value of 16.3 ± 12.6 km (median = 6.1 km). The average NND value calculated on mean values for each Annex II potential area was 1.5 ± 3.5 km (Online Resource 10-11).

New occurrences of Annex II species detected within SACs but not reported in the standard data forms corresponded to 316 km of river stretches. Almost half of them (54%) fell outside the N2KN. We found 21 unprotected patches over seven Annex II potential areas, with an average value of 3 ± 2.6 patches per area. Unprotected patches had a mean cumulative length of 24.6 ± 30.2 km (median = 12.4 km) for each Annex II potential area, with a mean patch density of 0.48 ± 0.35. Accounting for patch density, the mean value amounted to 8.2 ± 11.5 km (median = 1.8 km), whereas the average value of NND was 5.2 ± 3.9 km (Online Resource 12).

### eDNA Scoring

**Fig. 6:**
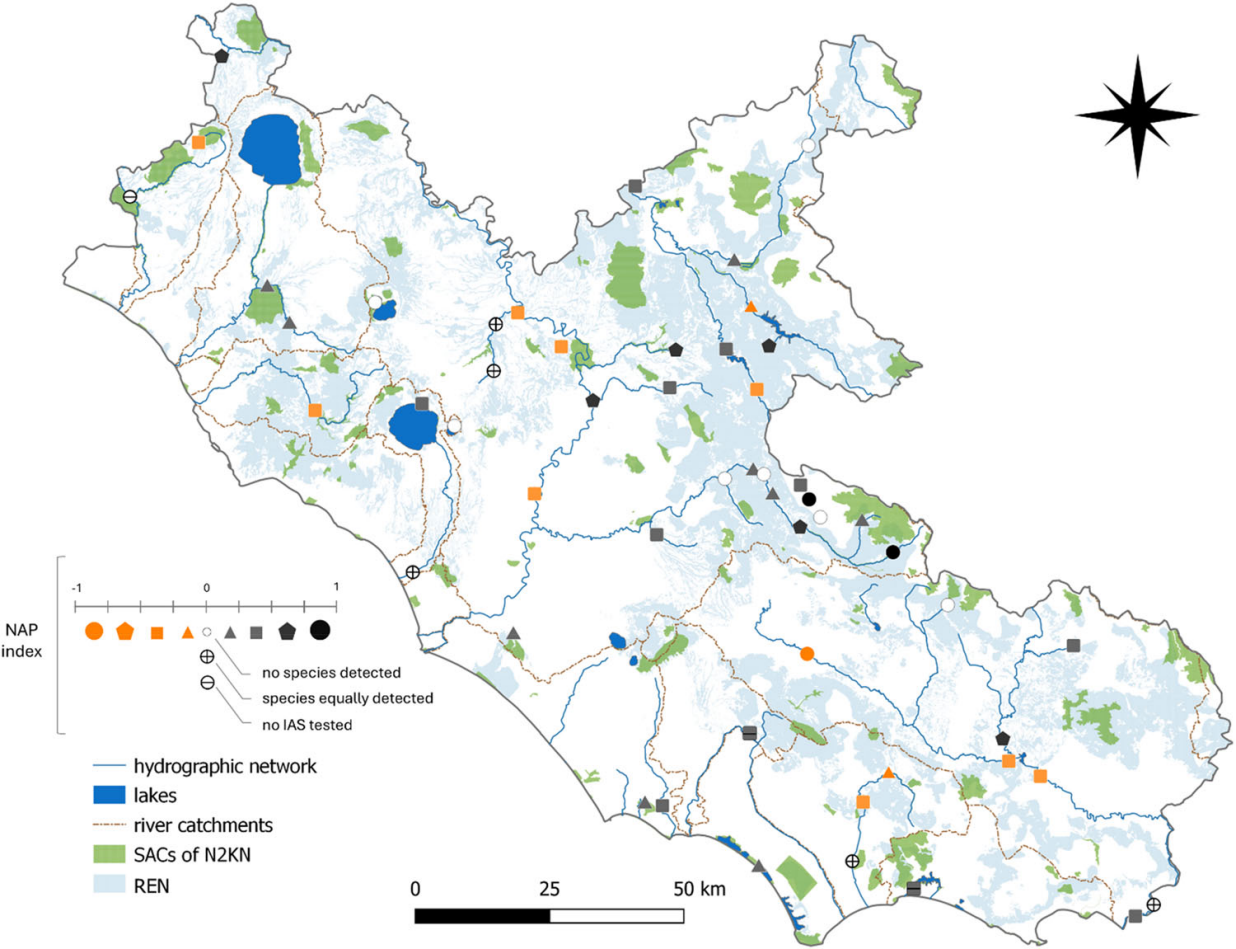
Native-Alien Prevalence values (NAP) ranging from -1 to 1, showing where native endangered species (NAP > 0, dark colours) and IAS (NAP < 0, orange colours) were respectively more detected with eDNA compared with the number of tested species. When NAP = 0, native endangered species and IAS ratios are equal due to either non-detection (white dots) or equal detection rates (crossed circles). Black circles or squares with a central dash indicate sites where IAS were not tested

The Native-Alien Prevalence index (NAP) ranged from –0.83 to 1 (Fig. 6; Online Resource 13). Specifically, 28 and 12 sites showed values > 0 (pristine sites) and < 0 (heavily colonized sites), respectively. The remaining 13 sites had a value NAP = 0 (both species occur at the same proportion). In five of these sites, native endangered species and IAS were detected in the same proportion, whereas at eight sites, no species were detected (Online Resource 14). Pristine areas with NAP > 0 were concentrated in the interior hills and mountains, whereas areas heavily invaded by IAS were mainly located in the lower plains of central and southern Latium. Vulnerable areas with NAP = 0 were mainly located along tributaries and small rivers (Fig. 6).

**Fig. 7:**
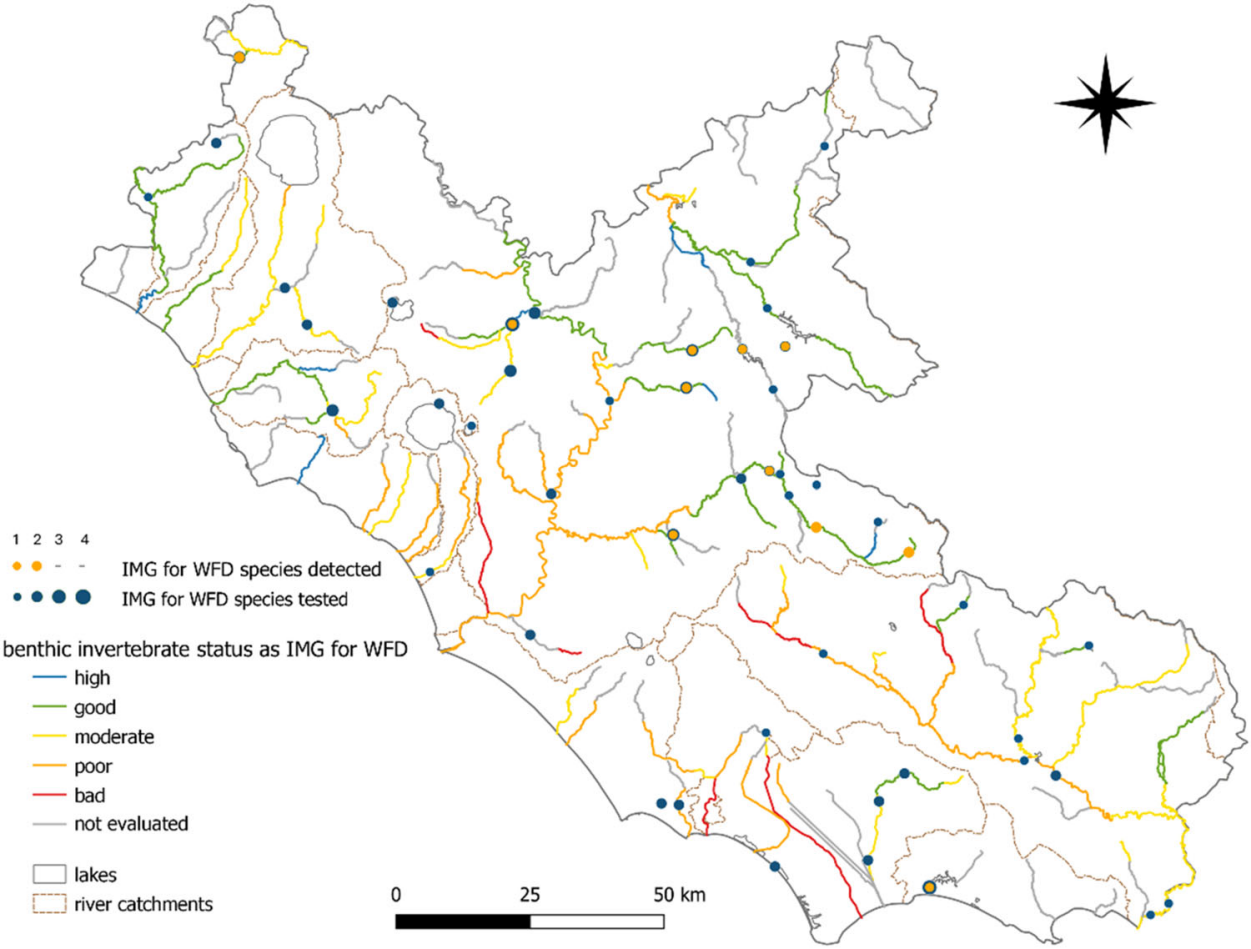
Details on locations of detections over tested sites for freshwater species used in WFD monitoring. Benthic species and their relative index as evaluated by the STAR_ICMi index in the Italian Monitoring Guidelines to assess the ecological status of water bodies for the Water Framework Directive (IMG for WFD) 2021-2023

**Fig. 8:**
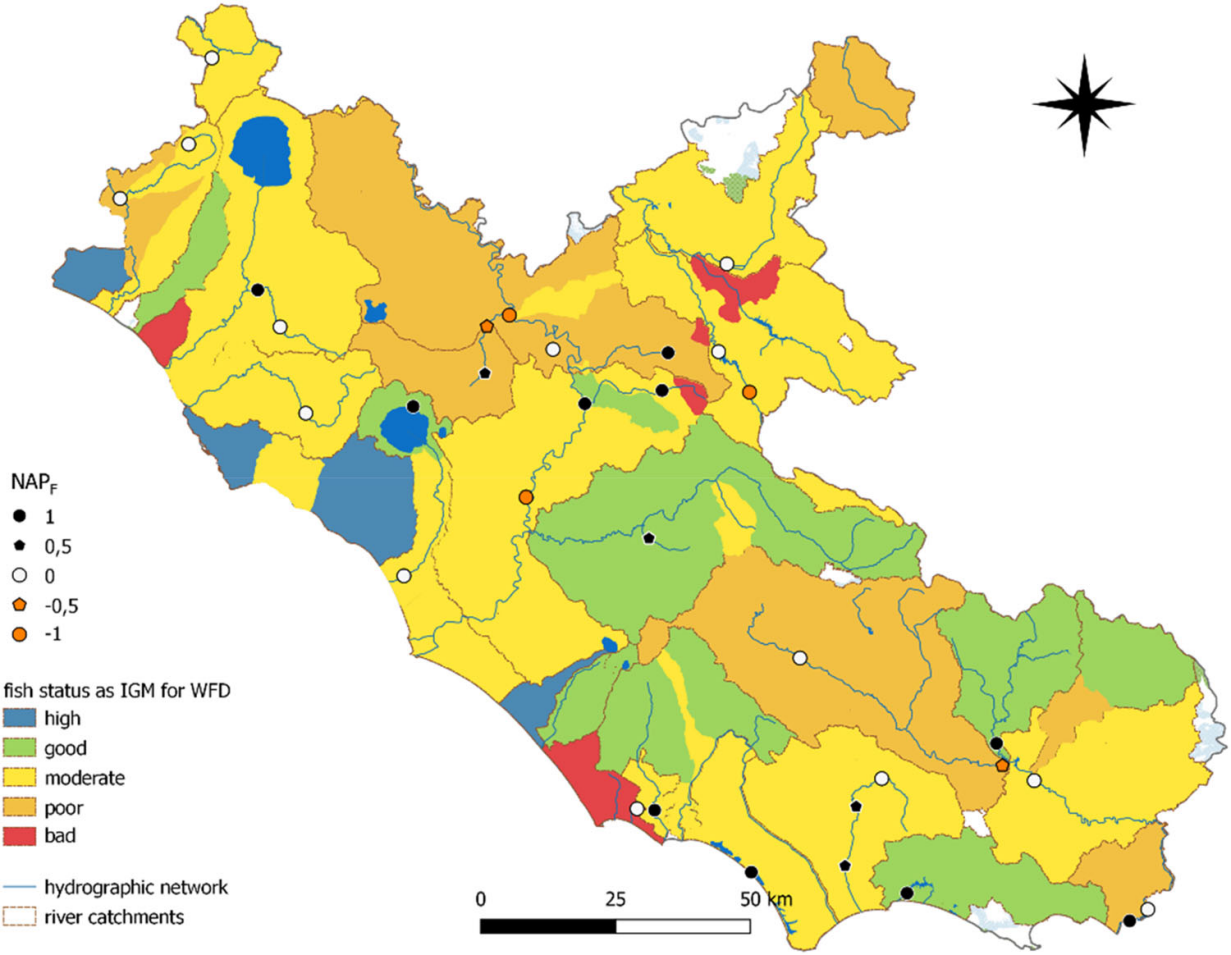
Details on locations of detections over tested sites for freshwater species used in WFD monitoring. Fish Native Alien Prevalence (NAP_F_) index with relative index as evaluated by the NISECI index in the Italian Monitoring Guidelines to assess the ecological status of water bodies for the Water Framework Directive (IMG for WFD) 2021-2023

Two benthic invertebrates included in WFD evaluations occurred at 11 sites (a total of 14 records) (Fig. 7). Most of these sites were located on inland rivers with a Good status for STAR_ICMI, one on a river classified as Moderate, and 4 on rivers Not Evaluated. Fish included in NISECI were detected at 32 sites, for a total of 37 occurrences. The NAP_F_ index ranged from –1 to 1 (Fig. 8; Online Resource 15). Specifically, 14 sites showed a NAP_F_ > 0, mostly located in the hills of the central region and along the southern coastline, and mostly on rivers evaluated as Good and Moderate status from NISECI assessments (Fig. 8). A NAP_F_ < 0 was detected for five sites, mainly located in the lower plains and near reservoirs, mostly on rivers classified as Moderate or Poor. The remaining 13 sites showed NAP_F_ = 0 (one site with no species detected and seven with only IAS or native species tested), and were located along Moderate and Poor rivers (Online Resource 15).

#### Prioritizing Protection Score (PPS)

**Fig. 9:**
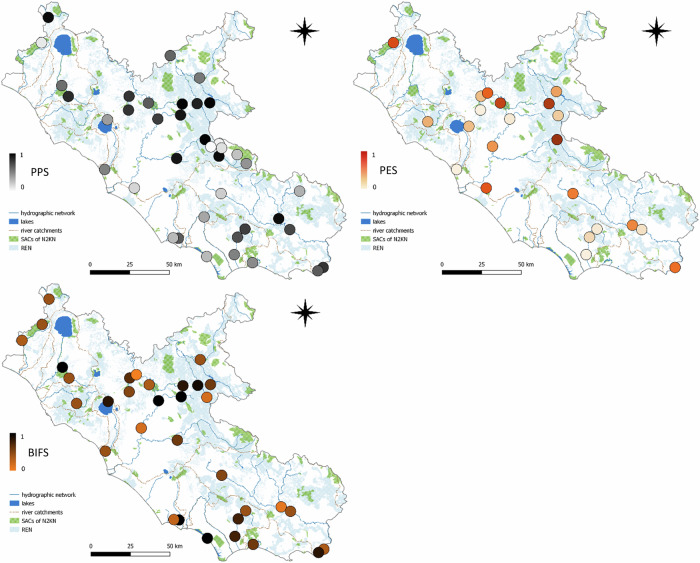
Priority areas for establishing new Special Areas of Conservation of Natura 2000 Network (SACs of N2KN) (PPS, Prioritizing Protection Score, top left), for launching eradication measures on IAS (PES, Prioritizing Eradication Score, top right), and where freshwater evaluations can benefit from eDNA surveys (BIFS, Benthic Invertebrates and Fish Score, bottom). REN= Regional Ecological Network

We found 39 sites with PPS ranging from 0.2 to 3.66 (x ± sd = 2.28 ± 1.02), requiring the establishment of new SACs within the N2KN (Online Resource 16). The five sites with the highest values were located along small tributaries in the central inland mountains (Fig. 9).

#### Prioritizing Eradication Score (PES)

PES was computed for the 22 sites of IAS occurrence. Values ranged from 0 to 4.34 (1.49 ± 1.35) (Online Resource 16). The five sites with the highest values were found in lowland valleys and overlapped with the highest values of PPE, i.e. areas where we detected Annex II species that could justify the establishment of new SACs (Fig. 9).

#### Benthic Invertebrates and Fish Score (BIFS)

The score was computed for 33 sites where the species contributing either to STAICMI or to NISECI were detected. BIFS values ranged from -2.2 to 3.3 (1.01 ± 1.53) (Online Resource 16). The five highest values were again obtained for the same areas showing high PPS and PES, and along the southern coastline (Fig. 9).

## Discussion

We demonstrated how a qPCR-based multi-species environmental DNA detection can support spatial conservation planning, help prioritize conservation measures, and reduce monitoring costs. Our results are consistent with Pascher et al (2022), who highlighted that multiple eDNA detection offers several advantages for effective long-term biodiversity monitoring of freshwater ecosystems, including tracking changes in species composition, early detection of biological threats such as invasive species, systematic detection of rare or cryptic species, and can be particularly applicable to the management of protected areas. A recent cost comparison between eDNA and traditional monitoring for the Eurasian otter by Giovacchini et al. (2026b) showed that eDNA-based monitoring reduced effective costs by 15%, despite traditional techniques yielding a slightly higher detection rate. In a multi-species framework, these savings scale with the number of target species, likely leading to a marked reduction in both survey time and monitoring costs.

Advantages offered by multi-species eDNA detection as a screening tool to facilitate early detection and eradication of aquatic invasive species in freshwater bodies have been recently evidenced by Flitcroft et al. (2025). These authors confirmed that a multi-species eDNA approach can meet multiple management objectives by looking for species across the plant and animal kingdoms sharing the same sample, revealing the sensitivity of eDNA for a multi-species community of IAS, especially efficient for fish, invertebrates, amphibians and submerged plants.

Among the most relevant findings for individual species were evidence of newly colonized biotopes by both native and alien species, contributing to upcoming EU reporting cycles. These include the return of the Eurasian otter (*Lutra lutra*) in the region after its extinction in the last century (Giovacchini et al., 2026b), the persistence of the endangered diadromous fish *Alosa fallax* in free-flowing rivers, coastal lakes, and even upstream of certain dammed stretches, and, in contrast, the still limited occurrence of Tuscanian chub *Squalius lucumonis* (Tancioni et al., 2^013^; Pompei et al., 2^018^; Carosi et al., 2^021^) (Online resources 1-7). Specifically, the return of the Eurasian otter in the region confirms the ongoing range expansion in the Italian peninsula (Giovacchini et al., 2^018^). New valuable occurrences of the endangered river mussel *Unio mancus* and white-clawed crayfish *Austropotamobius italicus* (Fureder et al., 2^010^; Lopes-Lima et al., 2^024^) suggest that the ecological status of the S. Vittorino Creek, Fondi outflowing channel, and Fosso della Foresta may be higher and should be re-assessed, as well as Treia river (Online resources 1-7, Fig. 7). Among IAS, the zebra mussel (*Dreissena polymorpha*) was recorded for the first time in the study area in the Tiber River basin. In fact, high densities of individuals were directly observed by one of the authors (SG) in two reservoirs used for angling, already heavily invaded by IAS fish (Grano 2022). Additionally, our results revealed the expansion of alien fish species such as the Padanian goby (*Padogobius bonelli*) and the topmouth gudgeon (*Pseudorasbora parva*). These findings confirm an ongoing trend of alien fish invasions in Central Italian rivers over the past decade, posing significant threats to native fish communities (Carosi et al., 2^021^; Sarrocco et al., 2^012^). These findings were also corroborated by recent field observations. For instance, the presence of the endangered twaite shad (*A. fallax*) was confirmed in the lower stretch of a river successfully assessed using eDNA (Ferri et al., 2024). Similarly, traditional field surveys revealed an unexpectedly broader distribution of the river mussel *U. mancus* (SG, unpublished data).

The assemblages of endangered species detected in headwater streams suggest that relict populations are persisting in remote mountainous areas, similar to the areas described in Grantham et al. (2020). However, our prioritization index (PPS) identified significant gaps in N2KN coverage, which should be addressed in the short term to move a step forward in the process of safeguarding these remnant populations. These gaps likely encompass habitats of high ecological integrity with minimal human disturbance (Foresta et al., 2016; Sallustio et al., 2017). Key areas requiring immediate conservation attention include sections of the Aniene River, Salto and Turano Lakes, and creeks flowing from the Sabine Hills. These sites are characterized by rare, endemic, and pollution-sensitive species such as *U. mancus, A. italicus, B. pachypus*, and *S. lucumonis*, together with secondary species of Community interest such as *Vertigo* sp. and *A. fallax*. These areas deserve either the designation of Special Areas of Conservation under the EU Habitats Directive 43/92/EC or the expansion of neighbouring N2KN sites.

A notable example is the Garigliano River, which marks the boundary between two administrative regions (Latium and Campania) and flows through intensively cultivated lands. Currently, only one riverbank is included within a Natura 2000 site (SAC, IT8010029). This study supports the establishment of the interregional Park of the Garigliano River proposed by the Regional Law of Latium 29/1997 but still not in place, thus providing effective conservation of this area through a coordinated management among regional authorities overseeing the riverbanks and the main river course. Such collaboration is crucial for protecting endangered species, including the recently rediscovered Eurasian otter (*Lutra lutra*), by surveillance to combat illegal activities and supervising the quality of undisturbed riparian and aquatic habitats (Valentim et al., 2^025^). Similarly, the expansion of Natura 2000 sites to include adjacent creeks and streams that border the protected areas would enhance conservation outcomes. For instance, the Amaseno River, which borders the Ausoni Mountains Natura 2000 site (IT6040043), remains unprotected despite its ecological significance. Rivers are frequently used as administrative boundaries, leading to their exclusion from conservation designations. Expanding existing protected areas to encompass river channels would facilitate the preservation of entire aquatic habitats with relatively minimal effort (Valentim et al., 2^025^).

The designation of new Special Areas of Conservation aligns with the long-term objectives of the European Green Deal and the Biodiversity Strategy for 2030, which aim to protect 30% of Europe’s land surface and freshwater ecosystems (Hermoso et al., 2022; Spiliopoulou et al., 2023). A key component of this initiative is the expansion of the existing 27,000 Natura 2000 sites. In fact, the establishment of protected areas is among the most effective conservation strategies for safeguarding biodiversity, particularly in wetlands (Chen et al., 2^023^). While land abandonment offers great opportunities for passive rewilding, achieving the 30% conservation target also should require the inclusion of productive landscapes (Müller et al., 2^020^). This challenge is less pronounced for freshwater ecosystems intersecting agricultural areas in seminatural contexts such as those located in South-Central Italy, as riverine networks and their associated vegetation belts are often underutilized by landowners. This scenario presents an opportunity for sustainable land-sharing approaches that minimize conflicts between land use and conservation objectives (Araújo and Alagador 2024).

The proposed Prioritizing Protection Score (PPS) and Prioritizing Eradication Score (PES) indicated a strong spatial overlap between priority areas for IAS management and key areas for the conservation of endangered species listed in the Habitats Directive. In these areas, immediate removal efforts are warranted to mitigate IAS impacts (Guerra et al., 2^018^). Being mainly located at higher altitudes, establishing IAS-free refugia would be particularly critical for those endangered species facing additional threats. e.g. from climate change (Johovic et al., 2^020^). Additionally, the removal of IAS should be prioritized within existing Special Areas of Conservation to ensure the effectiveness of the N2KN for biodiversity conservation (Baquero et al., 2^021^). This is the case for Latera Lake, where both *P. clarkii* and *Vertigo* sp. were found, underscoring the need for targeted eradication actions.

Key areas outside the N2KN requiring urgent intervention for IAS are found along the Aniene River, a main tributary of the Tiber river basin, where *B. dendrobatidis* was detected alongside *B. pachypus* and *A. italicus*. As the Tiber is the main river of Central Italy, future monitoring should focus on the area of occurrence of this fungus and prevent its spreading across the river basin to promote an effective implementation of both the Regulation on IAS and the WFD (Boon et al., 2^020^). In Turano Lake, two IAS, *F. limosus* and *D. polymorpha* co-occurred with *A. italicus* and *B. pachypus*, requiring urgent removal actions as these populations are still confined up to now.

Preventative actions should be undertaken in areas where no IAS were detected. These actions include stakeholder engagement, sociological surveys, and controls on fish restocking, fish farms, and aquaculture facilities, including reptile pet shops (Hermoso et al., 2^016^). Monitoring, especially eDNA-based, is also a fundamental part of the prevention process, as a rapid warning of the introduction of new IAS can promptly activate the eradication measures (Boon et al., 2^020^; Essl et al., 2^020^).

In this article, we showed that a qPCR eDNA approach allows the monitoring obligations of HD and IAS regulations to be fulfilled, and may also provide new useful information on species mandatorily monitored to assess the ecological status of single water bodies according to the WFD. The combined results could inform conservation policy on priority areas and optimize actions to preserve the native endangered biodiversity. Moreover, as the new record of a species listed in Annex II of HD may merit the designation of a SAC, this protected area can also be registered under the areas for which the water quality should be maintained or improved, as demanded by the WFD, for the protection of the species associated with water. This improves the efficacy of freshwater conservation, as often protected areas do not have enough resources dedicated to riverine management (Thieme et al., 2^012^). The WFD mandates management at the whole river catchment scale with adequate planning, thus creating interconnections and synergies with legislation addressing the protection of endangered species and IAS removal more widely and not only on specific sites (Birk et al., 2^012^; Kail et al., 2^023^). This could especially favour wide-ranging endangered freshwater species such as the Eurasian otter, catadromous and anadromous fish, and species sensitive to water quality (Hermoso et al., 2^016^, ^2022^).

Our multi-species eDNA-based approach revealed a finer level of information on bioindicator species that could contribute to the assessment of the ecological status of rivers. qPCR has already been demonstrated to be accurate for the detection of freshwater macroinvertebrates, and several efforts are underway to standardize eDNA methodologies and integrate them into the WFD evaluations (Leese et al., 2^018^). According to our findings, Blackman et al. (2024) recently showed that eDNA monitoring could improve the resolution of biotic indices used in WFD assessments and inform early warning systems for rapid changes in sensitive aquatic communities. Under the WFD, the ecological status of water bodies is determined by the status of the worst subordinate class (i.e., one-out-all-out rule). Consequently, the presence of IAS has the potential to influence ecological status classifications, given their well-documented impacts on biotic communities (Pont et al., 2^021^; Blancher et al., 2^022^; Macher et al., 2^025^).

This is particularly important because the contribution of IAS to the deterioration of the ecological status of a water body is often neglected. Even if it is recommended to include an evaluation of all IAS (i.e., vertebrates, invertebrates, aquatic plants) in the ecological status of a river body, currently only fish have gained attention from a few countries, incorporating the proportion of alien and native fish into fish-based assessments (e.g. in the Italian monitoring guidelines: Macchio et al., 2^017^). Alien fish represent a serious threat to freshwater habitats, and it is therefore crucial to evaluate the extent to which at least a part of the biotic community of the ecosystem (i.e., the freshwater fish communities) has been altered by IAS invasion. Further advances on this subject may be made in future, as the EU ECOSTAT Working Group has included it in their work programme. Maybe in the future these biological elements will be a standardized part of routine monitoring according to WFD guidelines (Vandekerkhove et al., 2^013^). In this context, there is room in future to further extend our qPCR eDNA multi-species monitoring approach to other benthic invertebrates, IAS and species included in HD Annexes to give a more exhaustive and integrated framework on the ecological status of freshwater bodies and the distribution of species of Community interest of specific territories (Hering et al., 2^018^).

### Limitations

Although our approach proved to be a cost effective and accurate method for the rapid and simultaneous detection of many species of Community interest, some limitations and bias should be considered when planning multi-species monitoring by using a qPCR single-species eDNA approach.

In standing waters having limited inflows, eDNA may be a valuable tool for assessing species occurrences. Molecular traces are well known to be less mobile under low-flow conditions and tend to settle within the water column (Burian et al., 2^021^). Owing to the fine spatial resolution of eDNA signals in such environments, eDNA-based approaches can support the identification of candidates for protected areas in lentic biotopes. In contrast, in running water, the downstream transport of eDNA can lead to false-positive detections. To address the uncertainty regarding the source of eDNA in lotic systems, we inferred the presence of species listed under the Habitats Directive (HD) within a range extending up to 10 km upstream (Burian et al., 2^021^; Sepulveda et al., 2^023^). As eDNA particles undergo rapid degradation in aquatic environments, qPCR-based eDNA usually guarantees reliable assessments of species assemblages at each site location (Dejean et al., 2^011^; Maruyama et al., 2^014^). Nevertheless, it is possible to partially limit the spatial uncertainty of eDNA occurrences by positioning sampling points in the headwaters. This strategy offers a cost-effective way to identify eDNA sources within the river network, and it meets the recommendations produced by complex modeling integration of hydrology and river morphology (Carraro et al., 2^021^). Indeed, in our study area, new occurrences were mainly located in headwaters, leading us to believe that our results accurately located the source of molecules belonging to new populations of native endangered species.

In addition, Pascher et al. (2022) highlighted several challenges that may limit the practical implementation of multi-species eDNA monitoring, including highly demanding data management and handling requirements, the need to assess method suitability across different biogeographical areas, and the gap between protected area management, laboratory scientists, and data analysis and interpretation. Overall, although eDNA approaches have strong potential to support biodiversity monitoring and assessment in the management of protected areas globally, standardized workflows from data collection to data evaluation and archiving still need to be developed and validated.

Finally, although abundance estimates are a key component of reporting obligations under the WFD, eDNA-based abundance estimates through eDNA are not yet sufficiently reliable, due to multiple sources of bias and noise introduced during sampling and processing into DNA-based datasets (Luo et al., 2^023^). Nevertheless, ongoing methodological and analytical advances are expected to progressively overcome these limitations.

## Conclusions

Multi-species eDNA survey based on quantitative PCR proved to be a powerful and cost-effective tool to satisfy monitoring obligations across a range of EU regulations aimed at biodiversity conservation of freshwater ecosystems, while also helping to prioritize protection measures. Consistent with other works (Pascher et al., 2^022^) our results demonstrate that this multi-species approach provides a cost-effective tool for the simultaneous detection of species of Community interest and the threats posed to them by alien species across large geographic areas.

The proposed scores PPS and PES enable the identification of priority areas for the management of invasive alien species under Regulation EU 1143/2014, as well as key areas for the conservation of native endangered species within existing and potential new Natura 2000 sites, while also allowing their trend to be monitored across time and space (Pascher et al., 2^022^). In this context, the identification of new potential Special Areas of Conservation is fully aligned with the long-term objectives of the European Green Deal and the Biodiversity Strategy for 2030, which aim to protect 30% of Europe’s land surface and freshwater ecosystems (Fernandez et al., 2^022^).

We also demonstrated how multi-species eDNA detections together with PPS and PPE indices can contribute to the implementation of river basin management plans, through which the Water Framework Directive is directly linked to the Habitats Directive. This approach may support the harmonization of national methods to assess the ecological status of water bodies, as required by the WFD, Member States and the EU ECOSTAT Working Group and highlight synergies between the WFD and HD that remain insufficiently explored.

These issues are particularly relevant for Italy, which hosts the most diverse freshwater fish community in Europe, the highest amount of endemism in Europe, and one of the most severe levels of invasion by alien species (Reyjol et al., 2^007^; Lorenzoni et al., 2^019^; Magliozzi et al., 2^020^; Milardi et al., 2^020^). Our results from a large-scale survey allow the identification of critical gaps in freshwater protected area coverage, providing an accurate mapping of risk of IAS invasions, and informing conservation strategies at both site and river catchment scale in these highly disturbed and vulnerable areas (Cortina and Boggia 2014; Hermoso et al., 2^015^; Johovic et al., 2^020^; Niz et al., 2^023^).

## Supplementary information

Supplementary information

## Data Availability

All data supporting the findings of this study are available within the paper and its Supplementary Information.
