# Supplementary material for: Conservation Planning and Reporting Implications of qPCR-based Multi-species eDNA Detection Under EU Environmental Regulations
Source: Environ Manage. 2026 Jun 6;76(6):211. doi: 10.1007/s00267-026-02503-3 (PMC13242467; doi:10.1007/s00267-026-02503-3)
Supplement: Supplementary file 1 — Supplementary information [file 267_2026_2503_MOESM1_ESM.pdf]

Supplementary Information

Conservation planning and reporting implications of qPCR-based multi-species eDNA detection under EU environmental regulations

Environmental Management

Giovacchini S. [1], Mirone E. [1], Bruno A. [2,3], Ramazzotti F. [2,3], Caprotti L. [2], Monaco P. [1], Di Febbraro M. [1], Manfrin C. [4], Jamwal P.S. [1], Belluardo F. [1], Galimberti A. [2,3], Loy A. [1,5]

[1] University of Molise, Environmetrix Lab, Contrada Fonte Lappone, Pesche (IS) 89090, Italy

[2] University of Milano-Bicocca, ZooPlantLab, piazza della Scienza 2, Milan 20126, Italy

[3] National Biodiversity Future Center, 90133, Palermo, Italy

[4] University of Trieste, Department of Life Sciences, via L. Giorgieri 5, Trieste 34127, Italy

[5] CNR-IRET, Montelibretti (RM) 00015, Italy

Corresponding author: Simone Giovacchini, University of Molise, s.giovacchini@studenti.unimol.it

Online resource 1. Molluscs – Distribution of eDNA occurrences in the study area of Latium region, and new presence cells according to the last HD and EU IAS Regulation Reporting or other published surveys (see main document).

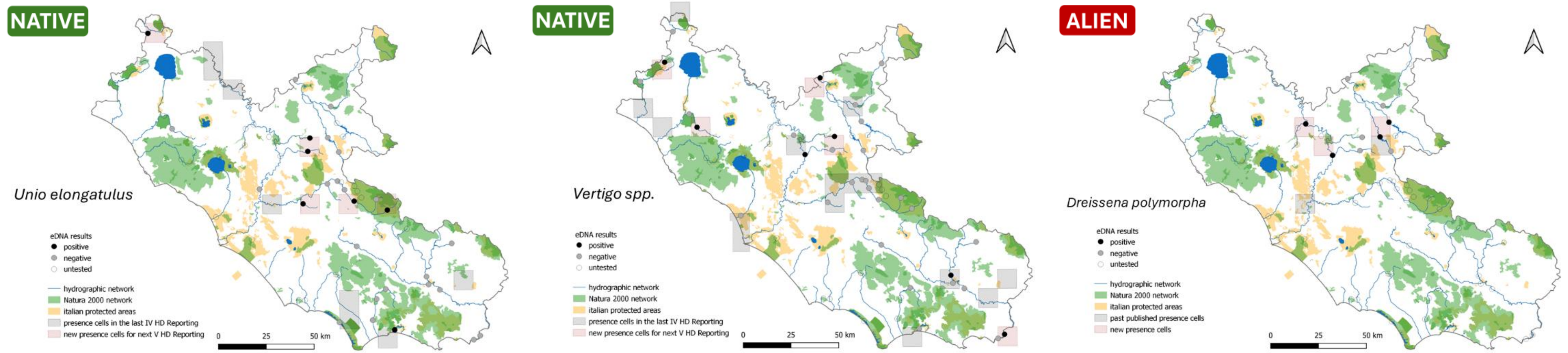

Online resource 2. Crustaceans – Distribution of eDNA occurrences in the study area of Latium region, and new presence cells according to the last HD and EU IAS Regulation Reporting or other published surveys (see main document).

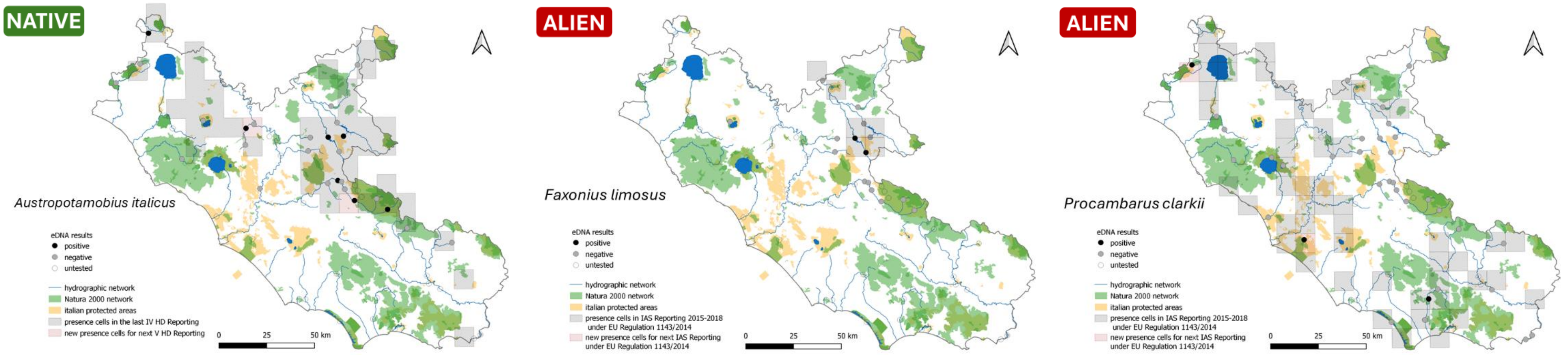

Online resource 3. Fish – Distribution of eDNA occurrences in the study area of Latium region, and new presence cells according to the last HD and EU IAS Regulation Reporting or other published surveys (see main document).

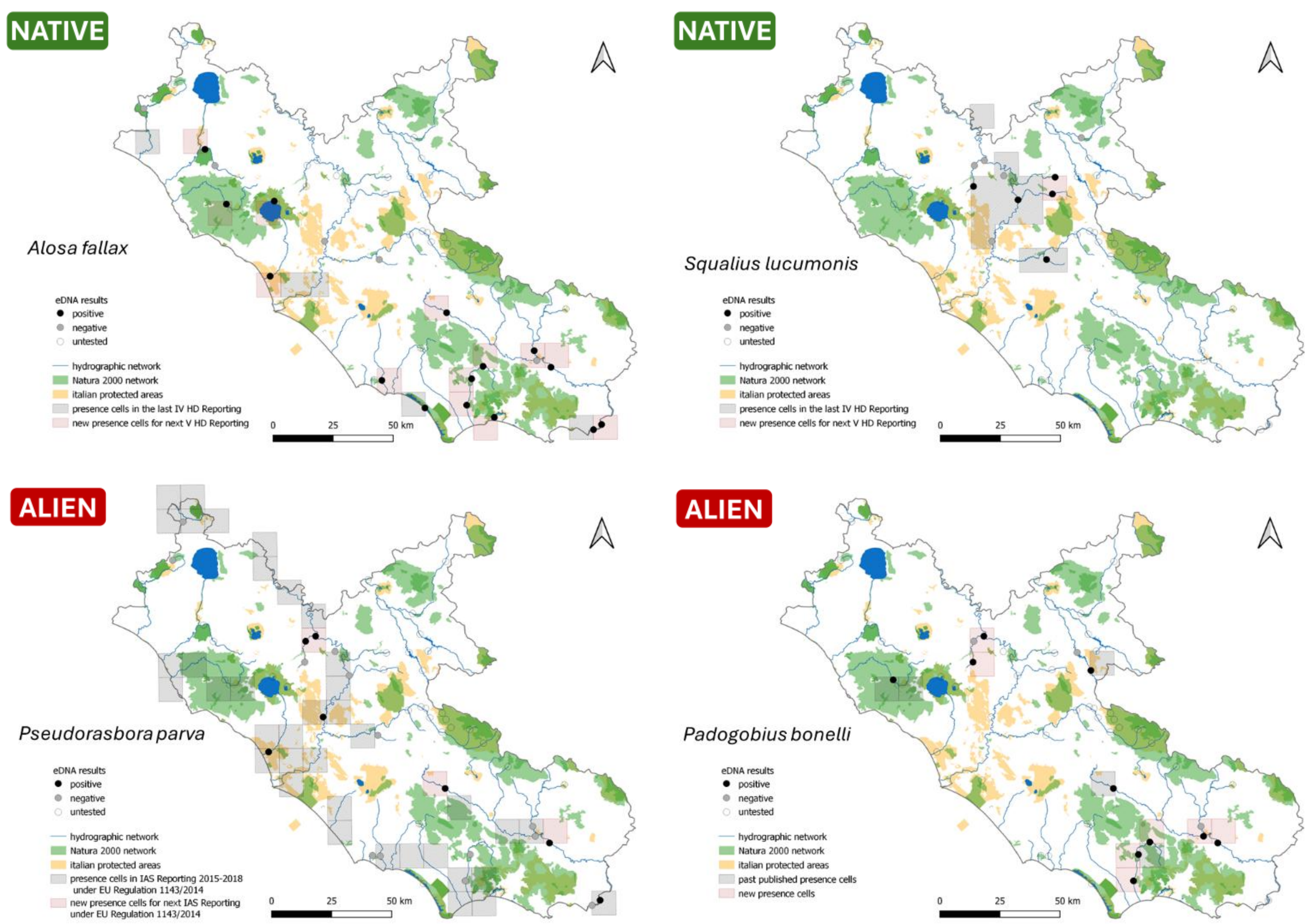

Online resource 4. Amphibians – Distribution of eDNA occurrences in the study area of Latium region, and new presence cells according to the last HD and EU IAS Regulation Reporting or other published surveys (see main document).

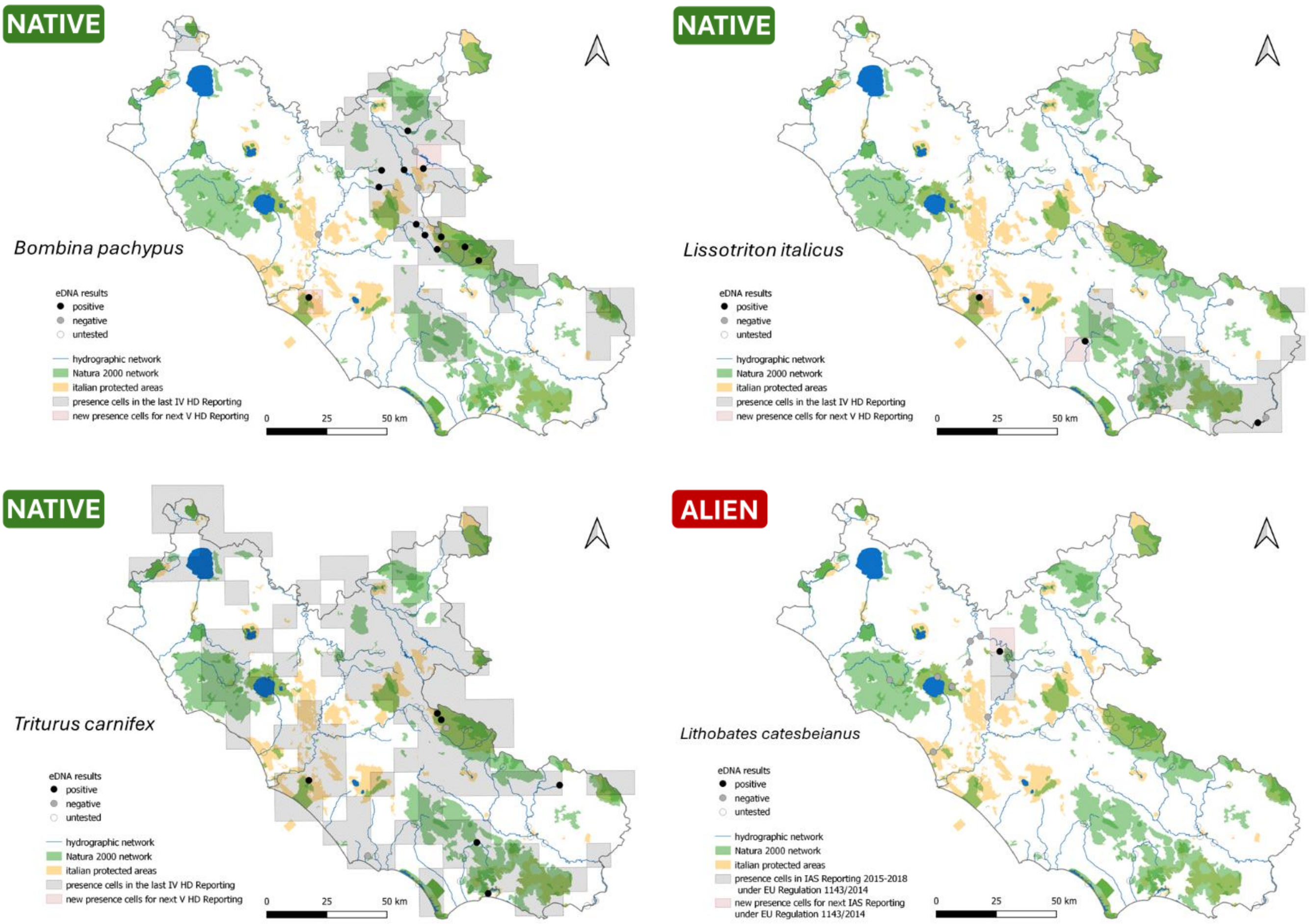

Online resource 5. Reptiles – Distribution of eDNA occurrences in the study area of Latium region, and new presence cells according to the last HD and EU IAS Regulation Reporting or other published surveys (see main document).

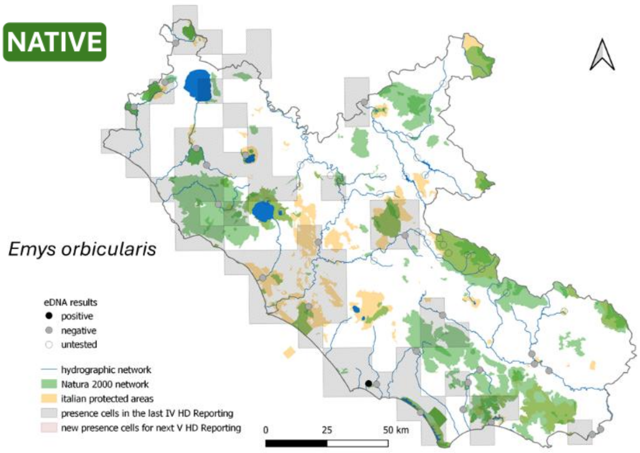

Online resource 6. Mammals – Distribution of eDNA occurrences in the study area of Latium region, and new presence cells according to the last HD and EU IAS Regulation Reporting or other published surveys (see main document).

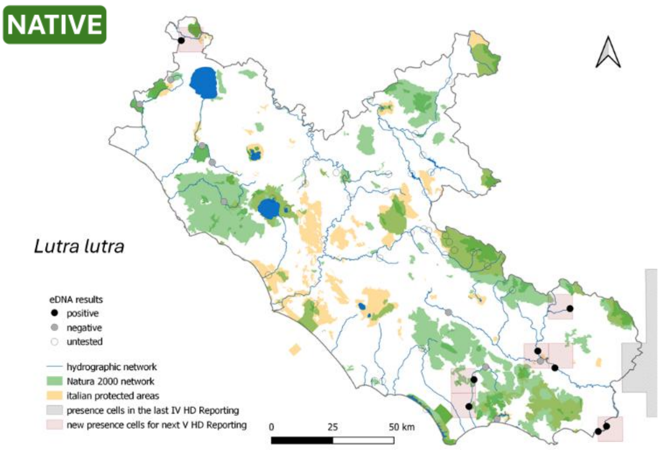

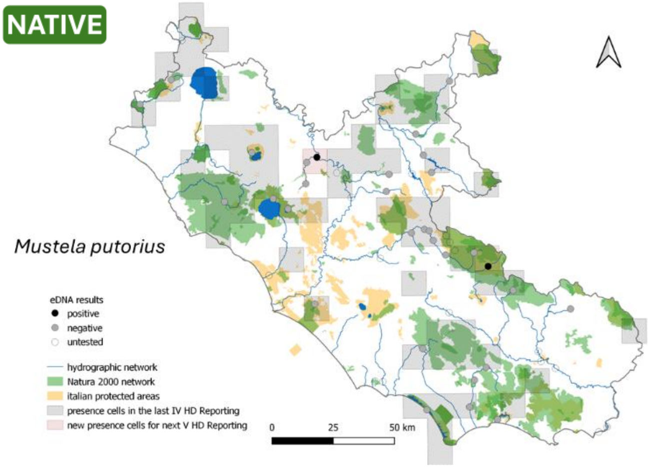

Online resource 7. Fungi – Distribution of eDNA occurrences in the study area of Latium region, and new presence cells according to the last HD and EU IAS Regulation Reporting or other published surveys (see main document).

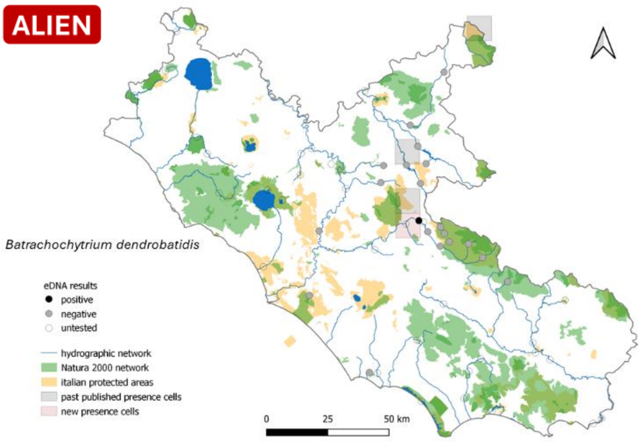

Online resource 8. Habitats Directive species found without Natura 2000 Network. Coordinates provided in WGS84 EPSG: 4326 georeferenced system.

Longitude	Latitude	Locality	HD Annex II species (N)	HD Annex II, IV, and V species (N)
13.5979139	41.4875261	San Giovanni Incarico	2	2
13.5186841	41.5528575	Ceprano	3	3
12.7532441	41.4688748	Latina	1	1
13.8336374	41.2626197	Santi Cosma e Damiano	3	3
13.792098	41.245147	Santi Cosma e Damiano	2	3
12.9416922	42.2282542	Rocca Sinibalda	2	2
12.8282994	42.2302169	Monteleone Sabino	3	4
12.8109656	42.1679005	Scandriglia	2	3
12.636599	42.151386	Fiano Romano	2	2
12.9899387	42.0220295	Roviano	2	2
13.0891749	41.9241672	Subiaco	2	3
12.216322	41.876915	Fiumicino	1	1
13.091545	41.711616	Anagni	1	1
12.766916	41.922676	Roma	1	2
12.4153373	42.208807	Mazzano Romano	1	1
12.4239651	42.2869587	Civita Castellana	1	1
11.9590775	42.2992023	Vetralla	1	1
11.8259389	42.7520841	Proceno	2	3
13.1694668	41.3615092	Pontinia	2	2
13.2602414	41.5039698	Prossedi	2	2
13.2003796	41.4594335	Roccasecca dei Volsci	2	2
13.0387416	42.2296686	Marcetelli	2	2
13.031233	41.9797335	Agosta	1	1

Online resource 9. Habitats Directive species found within Natura 2000 Network sites but not reported in the respective Standard Data Forms. Coordinates provided in WGS84 EPSG: 4326 georeferenced system.

Longitude	Latitude	Natura 2000 Network site	HD Annex II species (N)	HD Annex II, IV, and V species (N)	IAS (N)	IAS (species)
11.9116101	42.3622037	IT6010021 - Monte Romano	1	1	0	-
12.4361753	41.7647898	IT6030084 - Castel Porziano (Tenuta presidenziale)	1	1	1	*P. clarkii*
13.6874171	41.7025594	IT6050015 - Lago di Posta Fibreno	1	1	0	-
12.7531269	42.5079872	IT6020010 - Lago di Ventina	1	1	0	-
12.2513676	42.1583449	IT6030010 - Lago di Bracciano	1	1	0	-
12.969484	42.373852	IT6020012 - Piana di S. Vittorino - Sorgenti del Peschiera	1	1	0	-
11.7668453	42.6093847	IT6010012 - Lago di Mezzano	1	1	1	*P. clarkii*
12.961372	41.358138	IT6040012 - Laghi Fogliano, Monaci, Caprolace e Pantani dell'Inferno	1	1	0	-
13.303556	41.310775	IT6040010 - Lago di Fondi	1	2	0	-

Online resource 10. Details on locations sampling sites and relative ID.

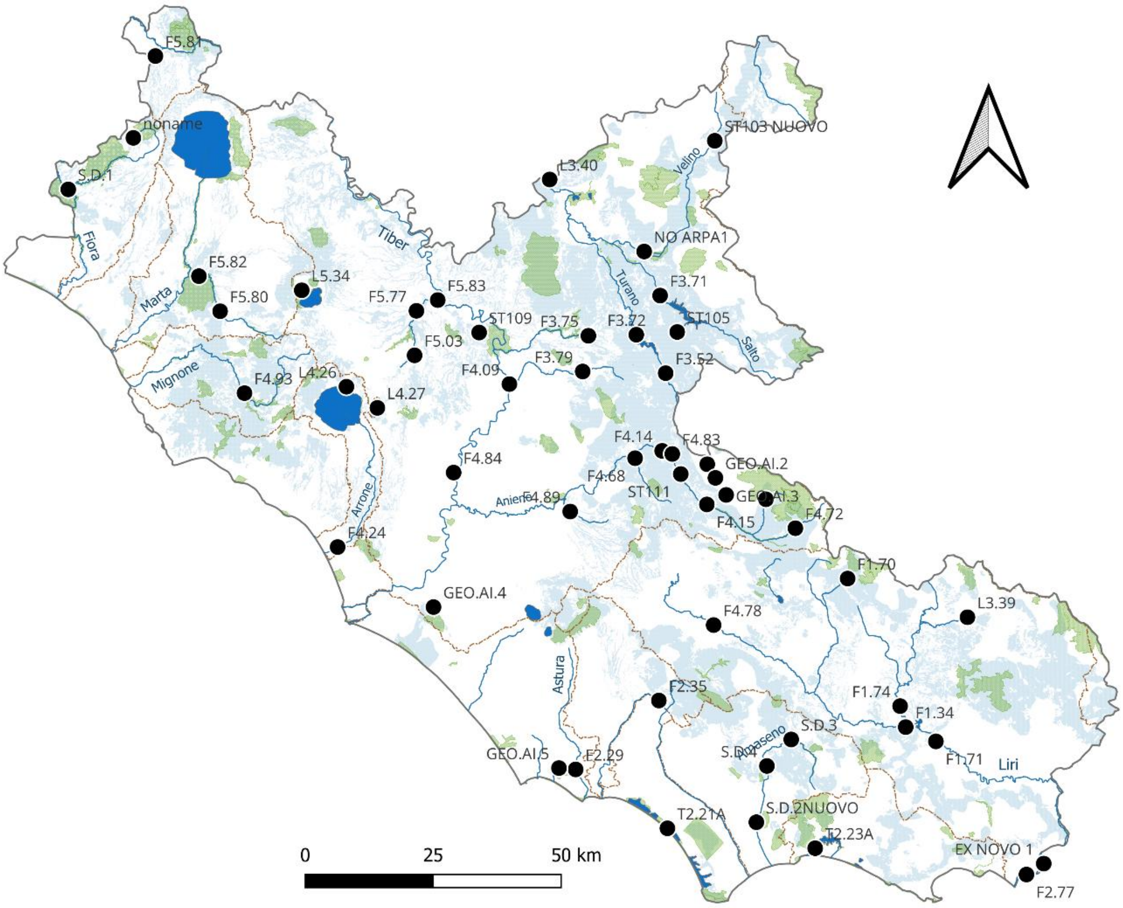

Online resource 11. Landscape metrics applied to 23 Annex II areas generated from the detections of Annex II species found without Natura 2000 network. NND: Nearest Neighbor Distance.

ID Annex II area	number of patches	respective site ID	cumulative length of stretches out of N2KN [km]	cumulative length of stretches in the area [km]	patch density	mean NND	sd NND
1	1	F5.81	11.408	11.408	1	0	0
2	1	F5.80	11.638	20.531	0.57	0	0
3	2	F5.77	69.040	80.427	0.86	901	0
3.1	1	F5.03	15.859	15.859	1	0	0
4	1	F4.24	21.097	21.097	1	0	0
5	1	ST109	2.425	2.425	1	0	0
6	3	F4.09	63.646	77.311	0.82	15486	2463
7	1	F3.75	16.791	16.791	1	0	0
8	1	F3.79	16.912	16.912	1	0	0
9	1	F3.72	13.395	13.395	1	0	0
10	1	ST105	2.020	2.020	1	0	0
11	1	F4.89	8.606	8.606	1	0	0
12	1	F4.14	38.687	42.419	0.91	0	0
13	3	F4.15	7.053	39.802	0.18	445	0
14	1	F4.78	39.079	39.393	0.99	0	0
15	1	F2.29	43.705	43.705	1	0	0
16	9	SD3	30.652	57.111	0.54	6162	609
18	1	F2.77	48.023	48.023	1	0	0
19	1	EXNOVO1	48.646	48.646	1	0	0
20	2	F1.71	78.416	81.034	0.97	2231	584
21	1	F1.74	59.111	59.111	1	0	0
22	3	SD2	20.772	24.481	0.85	2565	460
23	4	SD4	29.389	38.325	0.77	6152	846

x	1.82		30.276	35.166	0.88910866	1475	-
sd	1.8		22.422	24.299	0.20603167	3555	-
sum	42		696.370	808.832	-	-	-

Online resource 12. Landscape metrics applied to 7 Annex II areas generated from the detections of Annex II species found within Natura 2000 network but not already reported in respective standard data forms.

ID Annex II area	number of patches	respective site ID	cumulative length of stretches out of N2KN [km]	cumulative length of stretches in the area [km]	patch density	mean NND	sd NND
1	6	F5.82	82.743	93.395	0.89	2.132	1.372
2	7	L4.26	5.329	71.289	0.07	8.986	3.201
3	1	NOARPA1	12.404	24.342	0.51	5.600	0
4	1	GEO.AI.4	46.505	49.586	0.94	-	0
5	1	T2.21A	1.148	30.153	0.04	8.346	0
6	4	T2.23A	23.110	44.022	0.52	9.692	1.162
7	1	L3.39	1.319	3.421	0.39	1.958	707
x	3		24.651	45.172	0.48	6.119	-
sd	2.645751		30.206	30.085	0.352556	3.905	-
sum	21		172.558	316.208	-	-	-

Online resource 13. Index NAP calculated to assess the difference between two ratios of detection over tested species among protected species and IAS every sampling site.

ID sampling site	protected species detected (PSD)	protected species tested (PST)	IAS detected (IASD)	IAS tested (IAST)	PSD/PST (N_r)	IASD/IAST (A_r)	$N_r - A_r$ (NAP)
EX NOVO 1	3	6	1	2	0.5	0.5	0
F1.34	0	5	1	2	0	0.5	-0.5
F1.70	0	4	0	2	0	0	0
F1.71	2	5	2	3	0.4	0.66	-0.26
F1.74	3	5	0	2	0.6	0	0.6
F2.29	1	2	0	1	0.5	0	0.5
F2.35	1	3	0	0	0.33	0	0.33
F2.77	3	6	0	2	0.5	0	0.5
F3.52	0	4	2	5	0	0.4	-0.4
F3.71	0	4	1	4	0	0.25	-0.25
F3.72	2	3	2	5	0.66	0.4	0.26
F3.75	4	6	0	4	0.66	0	0.66
F3.79	3	6	0	4	0.5	0	0.5
F4.09	2	2	1	4	1	0.25	0.75
F4.14	2	5	1	3	0.4	0.33	0.66
F4.15	3	5	0	3	0.6	0	0.6
F4.24	1	3	1	3	0.33	0.33	0
F4.68	0	5	0	1	0	0	0
F4.72	4	5	0	3	0.8	0	0.8
F4.78	1	6	2	2	0.16	1	-0.83
F4.83	0	3	0	2	0	0	0
F4.84	0	6	1	3	0	0.33	-0.33
F4.89	2	4	0	1	0.5	0	0.5
F4.93	1	5	1	3	0.2	0.33	-0.13
F4.98	1	5	0	3	0.2	0	0.2
F5.03	1	3	1	3	0.33	0.33	0
F5.77	1	3	1	3	0.33	0.33	0
F5.80	1	5	0	1	0.2	0	0.2
F5.81	3	5	0	1	0.6	0	0.6
F5.82	1	4	0	1	0.25	0	0.25
F5.83	1	3	3	4	0.33	0.75	-0.42
GEO.AI.1	1	2	0	1	0.5	0	0.5
GEO.AI.2	2	2	0	1	1	0	1
GEO.AI.3	0	2	0	1	0	0	0
GEO.AI.4	3	5	1	2	0.6	0.5	0.1
GEO.AI.5	1	4	0	2	0.25	0	0.25
L3.39	2	7	0	1	0.28	0	0.28
L3.40	1	3	0	3	0.33	0	0.33
L4.26	1	2	0	2	0.5	0	0.5

L4.27	0	1	0	2	0	0	0
L5.34	0	3	0	1	0	0	0
NO ARPA1	1	6	0	4	0.16	0	0.16
noname	1	5	1	2	0.2	0.5	-0.3
S.D.1	0	6	0	0	0	0	0
S.D.2NUOVO	2	6	1	3	0.33	0.33	0
S.D.3	2	8	1	2	0.25	0.5	-0.25
S.D.4	2	6	2	3	0.33	0.66	-0.33
ST103 NUOVO	0	4	0	2	0	0	0
ST105	2	3	0	3	0.66	0	0.66
ST109	0	2	1	2	0	0.5	-0.5
ST111	1	5	0	3	0.2	0	0.2
T2.21A	1	4	0	1	0.25	0	0.25
T2.23A	3	8	0	0	0.375	0	0.375

Online resource 14. Details on locations of detections over tested sites for native protected species according to Habitats Directive (left) and IAS of Union Concern (right), composing N_r and A_r parameters of the index NAP.

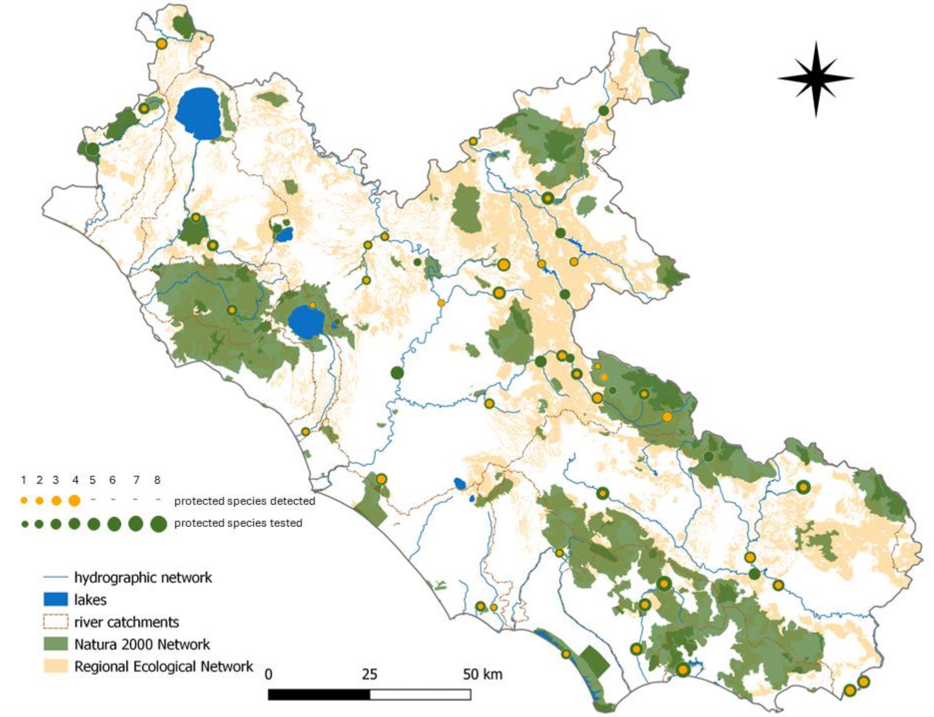

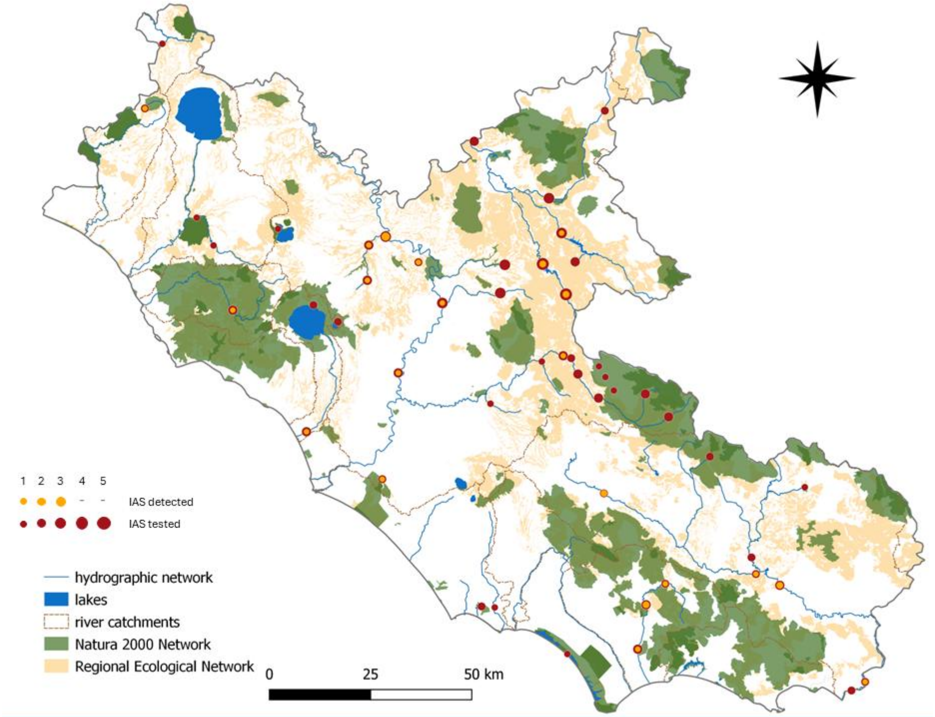

Online resource 15. Index of Native Alien Prevalence (NAP_f) calculated on fish species related to Water Framework Directive (WFD) to assess the difference between two ratios of detection over tested species among native fish (NF_r) and alien fish (AF_r) at each sampling site.

ID sampling site	native WFD fish species detected (NWFD)	native WFD fish species tested (NWFT)	alien WFD fish species detected (AWFD)	alien WFD fish species tested (AWFT)	NWFD / NWFT (NF_r)	AWFD / AWFT (AF_r)	$NF_r - AF_r$ (NAP_F)
EX NOVO 1	1	1	1	1	1	1	0
F1.34	0	1	1	2	0	0.5	- 0.5
F1.71	1	1	2	2	1	1	0
F1.74	1	1	0	2	1	0	1
F2.29	1	1	0	1	1	0	1
F2.77	1	1	0	1	1	0	1
F3.52	0	0	1	1	0	1	-1
F3.72	0	0	0	1	0	0	0
F3.75	1	1	0	0	1	0	1
F3.79	1	1	0	0	1	0	1
F4.09	1	1	0	1	1	0	1
F4.14	0	0	0	0	-	-	-
F4.15	0	0	0	0	-	-	-
F4.24	1	1	1	1	1	1	0
F4.72	0	0	0	0	-	-	-
F4.78	1	1	2	2	1	1	0
F4.84	0	2	1	1	0	1	-1
F4.89	1	2	0	1	0.5	0	0.5
F4.93	1	1	1	1	1	1	0
F5.03	1	1	1	2	1	0.5	0.5
F5.77	0	1	1	2	0	0.5	- 0.5
F5.80	0	1	0	0	0	0	0
F5.81	0	0	0	1	0	0	0
F5.82	1	1	0	0	1	0	1
F5.83	0	1	2	2	0	1	-1
GEO.AI.5	0	0	0	1	0	0	0
L4.26	1	1	0	0	1	0	1
NO ARPA1	0	1	0	0	0	0	0
noname	0	0	0	1	0	0	0
S.D.1	0	1	0	0	0	0	0
S.D.2NUOVO	1	1	1	2	1	0.5	0.5
S.D.3	1	1	1	1	1	1	0
S.D.4	1	1	1	2	1	0.5	0.5
ST105	0	0	0	0	-	-	-
ST109	0	1	0	1	0	0	0
T2.21A	1	1	0	0	1	0	1
T2.23A	1	1	0	0	1	0	1

Online resource 16. Table of scoring values for establishing new Natura 2000 sites, planning eradication strategies on IAS, and proposing new areas for species protection pursuant to WFD. HD= Habitats Directive. WFD= Water Framework Directive. N2KN= Natura 2000 network. REN= Regional Ecological Network. STAR_ICMi= Italian index to assess macrobenthic wildlife for WFD. NAP_F= Native Alien Prevalence Index on fish. NISECI= Italian index to assess fish communities for WFD.

		new Natura 2000 sites (HD)				IAS eradication planning (Reg. on IAS 1143/2014)				new Species protection areas (WFD)						
sampling site ID	index NAP	outside/not reported in N2KN	patch density	REN	scoring for new N2KN sites (PPS – Prioritization Protection Score)	within N2KN	direct threat at site	Potential threat in river catchment	scoring for IAS eradication (PES – Prioritization Eradication Score)	macro benthic species detected related to STAR_ICMi (A)	2021-23 STAR_ICMi (B)	NAP_F (C)	NISECI (2018-20) (D)	macrobenthos score (A*B) (E)	fish score (C*D+0.1) (F)	scoring for new WFD species protection areas (E+F) (FCS – Freshwater Conservation Score)
EX NOVO 1	0	2	1	1	3	1	0	1	2	0	-	0	2	-	0.2	0.2
F1.34	-0.5	-	-	-	-	0	0	1	1.5	0	-	-0.5	2	-	-1.2	-1.2
F1.70	0	-	-	-	-	-	-	-	-	-	-	-	-	-	-	-
F1.71	-0.26	2	0.97	1	2.74	0	0	0	0.26	0	-	0	3	-	0.3	0.3
F1.74	0.6	2	1	1	3.6	-	-	-	-	0	-	1	-	-	-	-
F2.29	0.5	2	1	0	2.5	-	-	-	-	0	-	1	3	-	3.3	3.3
F2.35	0.33	0	-	1	1.33	-	-	-	-	-	-	-	-	-	-	-
F2.77	0.5	2	1	0	2.5	-	-	-	-	0	-	1	2	-	2.2	2.2
F3.52	-0.4	-	-	-	-	0	0	0	0.4	0	-	-1	3	-	-1.2	-1.2
F3.71	-0.25	-	-	-	-	0	0	1	1.25	-	-	-	-	-	-	-
F3.72	0.26	2	1	1	3.26	0	2	2	3.74	1	1.5	0	3	1.5	1.8	3.3
F3.75	0.66	2	1	1	3.66	-	-	-	-	1	-	1	2	-	2.2	2.2
F3.79	0.5	2	1	1	3.5	-	-	-	-	1	-	1	3	-	3.3	3.3
F4.09	0.75	2	0.82	0	2.75	0	0	1	0.25	0	-	1	3	-	3.3	3.3
F4.14	0.66	2	0.91	1	3.66	0	2	3	4.34	1	-	-	-	-	-	-
F4.15	0.6	2	0.18	1	3.6	-	-	-	-	2	-	-	-	-	-	-
F4.24	0	2	1	0	2	0	0	0	0	0	-	0	3	-	0.3	0.3
F4.68	0	-	-	-	-	-	-	-	-	-	-	-	-	-	-	-
F4.72	0.8	0	-	1	1.8	-	-	-	-	2	-	-	-	-	-	-
F4.78	-0.83	2	0.99	0	1.17	0	0	1	1.83	0	-	0	2	-	1.2	1.2
F4.83	0	-	-	-	-	-	-	-	-	-	-	-	-	-	-	-
F4.84	-0.33	-	-	-	-	0	0	1	1.33	0	-	-1	3	-	-1.2	-1.2

F4.89	0.5	2	1	1	3.5	-	-	-	-	1	1.5	0.5	-	1.5	-	1.5
F4.93	-0.13	0	-	1		1	0	0	1.13	0	-	0	3	-	0.3	0.3
F4.98	0.2	0	-	1	1.2	-	-	-	-	-	-	-	-	-	-	-
F5.03	0	2	1	1	3	0	0	0	0	0	-	0.5	2	-	1.2	1.2
F5.77	0	2	0.86	1	3	0	0	1	1	1	3	-0.5	2	3	-1.2	1.8
F5.80	0.2	2	0.57	1	3.2	-	-	-	-	0	-	0	3	-	0.3	0.3
F5.81	0.6	2	1	1	3.6	-	-	-	-	2	-	0	3	-	0.3	0.3
F5.82	0.25	1	0.89	1	2.25	-	-	-	-	0	-	1	3	-	3.3	3.3
F5.83	-0.42	-	-	-	-	0	0	2	2.42	0	-	-1	2	-	-2.2	-2.2
GEO.AI. 1	0.5	0	-	0	0.5	-	-	-	-	-	-	-	-	-	-	-
GEO.AI. 2	1	0	-	0	1	-	-	-	-	-	-	-	-	-	-	-
GEO.AI. 3	0	-	-	-	-	-	-	-	-	-	-	-	-	-	-	-
GEO.AI. 4	0.1	1	0.94	0	1.1	1	0	2	2.9	-	-	-	-	-	-	-
GEO.AI. 5	0.25	0	-	1	1.25	-	-	-	-	0	-	0	1	-	0.1	0.1
L3.39	0.28	1	0.39	0	1.28	-	-	-	-	-	-	-	-	-	-	-
L3.40	0.33	1	-	1	2.33	-	-	-	-	-	-	-	-	-	-	-
L4.26	0.5	1	0.07	0	1.5	-	-	-	-	0	-	1	2	-	2.2	2.2
L4.27	0	-	-	-	-	1	0	0	1	-	-	-	-	-	-	-
L5.34	0	-	-	-	-	-	-	-	-	-	-	-	-	-	-	-
NO ARPA1	0.16	1	0.51	1	2.16	-	-	-	-	0	-	0	3	-	0.3	0.3
noname	-0.3	1	-	0	0.7	1	2	0	3.3	0	-	0	3	-	0.3	0.3
S.D.1	0	-	-	-	-	-	-	-	-	0	-	0	2	-	0.2	0.2
S.D.2NUOVO	0	2	0.85	0	2	0	0	0	0	0	-	0.5	3	-	1.8	1.8
S.D.3	-0.25	2	0.54	1	2.75	0	0	0	0.25	0	-	0	3	-	0.3	0.3
S.D.4	-0.33	2	0.77	1	2.67	0	0	0	0.33	0	-	0.5	3	-	1.8	1.8
ST103 NUOVO	0	-	-	-	-	-	-	-	-	-	-	-	-	-	-	-
ST105	0.66	2	1	1	3.66	-	-	-	-	1	1.5	-	-	1.5	-	1.5
ST109	-0.5	2	1	1	2.5	0	0	3	3.5	0	-	0	2	-	0.2	0.2
ST111	0.2	0	-	0	0.2	-	-	-	-	-	-	-	-	-	-	-
T2.21A	0.25	1	0.04	0	1.25	-	-	-	-	0	-	1	3	-	3.3	3.3
T2.23A	0.375	1	0.52	0	1.375	-	-	-	-	1	1.5	1	-	1.5	-	1.5
